# Optimization of universal allogeneic CAR-T cells combining CRISPR and transposon-based technologies for treatment of acute myeloid leukemia

**DOI:** 10.3389/fimmu.2023.1270843

**Published:** 2023-09-19

**Authors:** Cristina Calviño, Candela Ceballos, Ana Alfonso, Patricia Jauregui, Maria E. Calleja-Cervantes, Patxi San Martin-Uriz, Paula Rodriguez-Marquez, Angel Martin-Mallo, Elena Iglesias, Gloria Abizanda, Saray Rodriguez-Diaz, Rebeca Martinez-Turrillas, Jorge Illarramendi, Maria C. Viguria, Margarita Redondo, Jose Rifon, Sara Villar, Juan J. Lasarte, Susana Inoges, Ascension Lopez-Diaz de Cerio, Mikel Hernaez, Felipe Prosper, Juan R. Rodriguez-Madoz

**Affiliations:** ^1^ Hematology and Cell Therapy Department, Clinica Universidad de Navarra, IdiSNA, Pamplona, Spain; ^2^ Hematology Department, Hospital Universitario de Navarra, IdiSNA, Pamplona, Spain; ^3^ Centro de Investigacion Biomedica en Red de Cancer (CIBERONC), Madrid, Spain; ^4^ Hemato-Oncology Program, Cima Universidad de Navarra, IdiSNA, Pamplona, Spain; ^5^ Computational Biology Program, Cima Universidad de Navarra, IdiSNA, Pamplona, Spain; ^6^ Immunology and Immunotherapy Program, Cima Universidad de Navarra, IdiSNA, Pamplona, Spain; ^7^ Cancer Center Clinica Universidad de Navarra (CCUN), Pamplona, Spain; ^8^ Immunology and Immunotherapy Department, Clinica Universidad de Navarra, Pamplona, Spain; ^9^ Data Science and Artificial Intelligence Institute (DATAI), Universidad de Navarra, Pamplona, Spain

**Keywords:** allogeneic CAR-T, CRISPR, transposon, AML, transcriptomics (RNA sequencing)

## Abstract

Despite the potential of CAR-T therapies for hematological malignancies, their efficacy in patients with relapse and refractory Acute Myeloid Leukemia has been limited. The aim of our study has been to develop and manufacture a CAR-T cell product that addresses some of the current limitations. We initially compared the phenotype of T cells from AML patients and healthy young and elderly controls. This analysis showed that T cells from AML patients displayed a predominantly effector phenotype, with increased expression of activation (CD69 and HLA-DR) and exhaustion markers (PD1 and LAG3), in contrast to the enriched memory phenotype observed in healthy donors. This differentiated and more exhausted phenotype was also observed, and corroborated by transcriptomic analyses, in CAR-T cells from AML patients engineered with an optimized CAR construct targeting CD33, resulting in a decreased *in vivo* antitumoral efficacy evaluated in xenograft AML models. To overcome some of these limitations we have combined CRISPR-based genome editing technologies with virus-free gene-transfer strategies using *Sleeping Beauty* transposons, to generate CAR-T cells depleted of HLA-I and TCR complexes (HLA-I^KO^/TCR^KO^ CAR-T cells) for allogeneic approaches. Our optimized protocol allows one-step generation of edited CAR-T cells that show a similar phenotypic profile to non-edited CAR-T cells, with equivalent *in vitro* and *in vivo* antitumoral efficacy. Moreover, genomic analysis of edited CAR-T cells revealed a safe integration profile of the vector, with no preferences for specific genomic regions, with highly specific editing of the HLA-I and TCR, without significant off-target sites. Finally, the production of edited CAR-T cells at a larger scale allowed the generation and selection of enough HLA-I^KO^/TCR^KO^ CAR-T cells that would be compatible with clinical applications. In summary, our results demonstrate that CAR-T cells from AML patients, although functional, present phenotypic and functional features that could compromise their antitumoral efficacy, compared to CAR-T cells from healthy donors. The combination of CRISPR technologies with transposon-based delivery strategies allows the generation of HLA-I^KO^/TCR^KO^ CAR-T cells, compatible with allogeneic approaches, that would represent a promising option for AML treatment.

## Introduction

Adoptive immunotherapy using T cells engineered with Chimeric Antigen Receptors (CAR-T cells) has emerged as a promising therapeutic option for several B cell malignancies. The impressive results with CAR-T cells targeting CD19 and BCMA have led to the approval by the FDA and the EMA of several CAR-T cell products for refractory cell precursor acute lymphoblastic leukemia and large B cell lymphoma (1–4). Nevertheless, the use of CAR-T cell therapy for other hematological malignancies, and in particular for acute myeloid leukemia (AML), still presents specific challenges that hamper their efficacy and limit their implementation (5). One of the main biological barriers for CAR-T therapies in AML is the absence of AML-specific antigens. Most of the cell surface antigens present in AML blasts (CD33, CD123, or CLL1) are also present in normal hematopoietic, representing a safety concern, since a prolonged myeloablation would be ultimately fatal. Thus, several strategies to prevent the risk of bone marrow failure after CAR-T therapy have been proposed, including the limitation of CAR-T cell persistence, by the inclusion of safety switches, or the identification of neoantigens specific for AML blasts (5). Another interesting approach, currently under clinical evaluation, is the generation of leukemia-specific antigen by deleting CD33 from normal hematopoietic stem and progenitor cells, thereby enabling specific targeting of AML with CD33-CAR-T cells, since hematopoietic system would be resistant to CD33-targeted therapy (6). Nevertheless, most of the current CAR-T therapies under clinical evaluation are directed against CD33 or CD123, reporting antitumoral responses in some cases (7), although more complete clinical results have yet to be published. In addition, the dependency on patient-specific T cells for autologous approaches, particularly from heavily treated patients, may lead to inadequate T cell numbers, suboptimal CAR-T cell functions, and unsuccessful CAR-T cell production (8). However, there is a lack of studies performing a detailed and deep characterization of AML patient-derived CAR-T cells with a direct comparison to CAR-T cells from healthy donors.

The use of allogeneic CAR-T cells could overcome some of the limitations of autologous patient-specific CAR-T cells (9, 10). However, allogeneic cells may trigger graft-versus- host disease (GvHD) that would compromise therapeutic safety (11, 12). Since TCR ablation prevents GvHD, several strategies for endogenous TCR inactivation in CAR-T cells have been reported (9, 13). One of the most promising approaches relies on the use of TALEN and/or CRISPR technologies for TCR disruption (14, 15), which can be combined with different viral and non-viral vectors for the generation of allogeneic CAR-T cells (16–18). Despite allogeneic CAR-T cells can be successfully manufactured for therapeutic applications, there is an unmet need to overcome several limitations related to complex manufacturing procedures or the use of viral vectors, that would allow a cost-effective and safer generation of allogeneic CAR-T cell products. In this sense, the use of non-viral vectors based on *Sleeping Beauty* transposon systems for CAR delivery has emerged as a promising option that offers a number of advantages, including larger cargo capacity, reduced manufacturing complexity and costs, and safer integration profiles compared with integrating viral vectors (19–22). Nevertheless, the combination of *Sleeping Beauty* transposon systems with CRISPR technologies for the manufacturing of allogeneic CAR-T cells has not been extensively explored (18).

In this work, we have performed a deep phenotypic, transcriptomic, and functional characterization of T and CAR-T cells from AML patients that resulted in the identification of specific features that could compromise their antitumoral efficacy, compared to CAR-T cells from healthy donors. To overcome some of these limitations we have combined CRISPR-based genome editing technologies with virus-free gene-transfer strategies using *Sleeping Beauty* transposons, to generate allogeneic CAR-T cells targeting CD33. Our optimized protocol allows the generation of fully functional HLA-I^KO^/TCR^KO^ CD33-CAR-T cells in conditions compatible with clinical applications. These allogeneic CD33-CAR-T cells would represent a promising option for AML treatment, especially for therapeutic approaches in R/R AML patients where CAR-T cells are used to reduce tumor burden as bridging therapy prior to allogeneic stem cell transplantation.

## Materials and methods

### Patient’s samples and cell lines

Peripheral blood samples were obtained from patients with diagnosed AML as well as from young (below 30) and aged-matched healthy donors. Sample collection was conducted in accordance with the principles of the Declaration of Helsinki and with the approval of the Research Ethical Committee of the University of Navarra. All subjects provided written informed consent. Jurkat-TPR cells (kindly provided by Dr. P. Steinberg; Medical University of Vienna) were cultured in RPMI 1640 (Lonza) supplemented with 10% FBS (Gibco). MOLM-13 was cultured in RPMI 1640 supplemented with 20% FBS. HEK293T cells were cultured in DMEM supplemented with 10% FBS. All media were supplemented with 1% penicillin/streptomycin (Gibco) and 1% L-Glutamine (Gibco). All cell lines were maintained at 37°C in 5% CO_2_.

### Lentiviral vector construction and virus preparation

A third-generation self-inactivating lentiviral vector (pCCL) was used to express under the EF1a promoter second-generation CAR constructs targeting CD33, derived from M195 (23) or my96 (24) antibodies. CAR structure comprised the single-chain variable fragment (scFv), a panel of different hinge regions derived from CD8a or IgG4, a CD8 transmembrane domain, 4-1BB or CD28 co-stimulatory domain, and CD3ζ endodomain (**Figure S1A**). All constructs included an huEGFRt transduction marker separated from the CAR gene by a viral 2A sequence. Lentiviral vectors were produced in HEK293T cells following standard procedures. Briefly, 6×10^6^ cells were co-transfected with LV vector along with pMDLg/pRRE (Gag/Pol), pRSVRev, and pMD2.G (VSVG envelope) packaging plasmid using Lipofectamine 2000 (Invitrogen). Supernatants were collected 40h after transfection, filtered, concentrated using Lenti-X Concentrator (Takara) following manufacturer specifications, and stored at -80°C until use.

### Analysis of CAR signaling in Jurkat-TPR

Jurkat-TPR cells, transduced at MOI of 1 with CAR constructs targeting CD33 were co-cultured in triplicate with MOLM-13 cells, at a 1:1 effector to tumor cell ratio. Non-transduced Jurkat-TPR cells or transduced with a previously described CAR construct targeting CD19 (25) were used as control. Activation of the NFAT, NF-κB, and AP-1 pathways was quantified before and 24h after co-culture with tumor cells measuring eGFP, eCFP, and mCherry emissions respectively, using a CytoFLEX LX Flow Cytometer (Beckman Coulter) (**Figure S1B**).

### CAR-T cell generation

Peripheral Blood Mononuclear Cells (PBMCs) were isolated with Ficoll-Paque and CD4^+^ and CD8^+^ T cells were selected using CD4 and CD8 MicroBeads (Miltenyi Biotec) in the AutoMACS Pro Separator (Miltenyi Biotec). Isolated T cells were activated with 10 µl/ml T cell TransAct (Miltenyi Biotec) for 48h and infected with the CAR lentiviral vector at MOI 2 with 10 µl/ml of LentiBoost (Sirion Biotech). CAR-T cells were expanded during 10-12 days in RPMI 1640 culture medium supplemented with 3% human serum (Sigma), 1% penicillin/streptomycin, and 625 IU/ml of human IL-7 and 85 IU/ml of human IL-15 (Miltenyi Biotec). CAR-T cells were counted, and the concentration was adjusted to 1×10^6^ cells/ml every two days.

### Flow cytometry

Phenotypic characterization of T cells and CAR-T cells was performed at day 0 and 14 of the production, respectively. All antibodies were purchased from Biolegend unless otherwise stated (**Table S1**). Data was acquired on a BD FACSCanto II (BD Biosciences) and analyzed using the FlowJo Software version 10 (Tree Star).

### Cytotoxicity assay and cytokine production

Cytotoxicity was determined using MOLM-13-GFPLuc as target tumor cells. Briefly, MOLM-13-GFPLuc cells were cultured with CAR-T cells at different ratios in RPMI 1640 culture medium, supplemented with 3% human serum (Sigma) and 1% penicillin/streptomycin in Nunc™ 96-Well Round Bottom plates (ThermoFisher Scientific). After 24h, luminescence was measured using the Bright-Glo™ Luciferase Assay System (Promega) according to the manufacturer’s instructions. IFN-γ cytokine production was quantified using BD™ Immunoassay ELISA reagents (BD Biosciences) following manufacturer protocol.

### Continuous repeated stimulation

CAR-T cells were co-cultured with irradiated MOLM-13-GFPLuc tumor cells for 21 days. Briefly, 1×10^6^ cells MOLM-13-GFPLuc were irradiated at 54 Gy to prevent tumor growth, and co-cultured with CAR-T cells, at a 1:1 effector: target ratio of cells, in RPMI culture medium. Every three days, CAR T cells were counted and irradiated tumoral cells were added at a 1:1 ratio. On day 21, the phenotype of CAR-T cells was studied.

### *In vivo* experiments

All experimental procedures were approved by the Ethics Committee of the University of Navarra and the Institute of Public Health of Navarra according to European Council Guidelines. NOD-SCID-Il2rg^−/−^ (NSG) mice were purchased from The Jackson Laboratory (JAX) and bred and maintained in-house in a pathogen-free facility. Eight-to-twelve-week-old male or female mice were irradiated at 1.5 Gy at day -1 and 5×10^4^ MOLM-13-GFPLuc cells were intravenously injected the following day. Mice were randomized to ensure equal pre-treatment tumor burden before CAR-T cell treatment. At day 4 mice received i.v. injection of 3×10^6^ CAR-T cells. Mice were humanely euthanized when mice demonstrated signs of morbidity and/or hindlimb paralysis.

### RNA-sequencing and bioinformatics analysis

RNA-seq was performed following the MARS-seq protocol adapted for bulk RNA-seq (26, 27) with minor modifications. RNA-seq libraries quantification was done with Qubit 3.0 Fluorometer (Life Technologies) and size profiles were examined using Agilent’s 4200 TapeStation System. Libraries were sequenced in an Illumina NextSeq500 at a sequence depth of 10 million reads per sample. Samples were aligned to the Human genome (GRCh38) with STAR (v2.6.1). Gene expression was quantified with quant3p (github.com/ctlab/quant3p). Downstream analyses were performed in R (v3.6.2). Data transformation, normalization, and differential gene expression analysis were performed with DESeq2. Genes were considered adjusted p value (p-adj)<0.05 and absolute log transformed fold-change (|log2FC|)>1, unless otherwise indicated. vst expression values were used for data visualization and unsupervised analysis. Stem cell memory, T cell activation, and exhaustion gene signatures used in this work were obtained from previous publications (28–31) (**Table S2**). The normalized gene expression matrix was used to discover the disrupted genes after stimulation of AML CAR-T cells in comparison to adult and senior CAR-T cells. These genes were found using the maSigPro package (version 1.72.0) (32) which applies a negative binomial model to the expression distribution and adjusts the false discovery rate using the Benjamini and Hochberg procedure. The degree of polynomial regression in this study was set to 2, and the two ways forward elimination algorithm was used to perform stepwise regression to select genes with alpha equal to 0.05. To extract the significant genes upon stimulation the following settings were used: min.obs=2, and rsq=0.7.

### sgRNA design and *in vitro* evaluation

sgRNAs targeting exon 1 of the beta-2-microglobulin (B2M) gene and exon 1 of T-cell receptor α constant (TRAC) locus were designed and selected as described previously (33) using Benchling software (www.benchling.com). Sequences for B2M and TRAC sgRNAs can be found in **Table S3**. *In vitro* cleavage efficiency was evaluated by TIDE (6) after transfection of *Streptococcus pyogenes* Cas9 (SpCas9) and sgRNA ribonucleoprotein complexes (RNP) in the Jurkat cell line. SpCas9 protein and sgRNAs were purchased from IDT. 2x10^6^ Jurkat cells were electroporated with 61 pmol of RNP (ratio 1:1 Cas9:sgRNA) using the SE Cell Line 4D-Nucleofector Kit and the CL120 program on a 4D-Nucleofector System (Lonza) according to manufacturer’s instructions. Genomic DNA was isolated with NucleoSpin Tissue for DNA extraction kit (Macherey-Nagel) 72h after electroporation and subjected to targeted PCR amplification using primer described in **Table S4**. Indel percentage was calculated using the TIDE webtool (https://tide.nki.nl).

### Preparation of MC DNA and SB100X mRNA

Minicircle (MC) encoding CD33 targeting CAR was generated from parental pT2 plasmids by PlasmidFactory, using site-specific recombination and purified by affinity chromatography. Poly(A)-tailed ARCA-capped SB100X mRNA was produced by *in vitro* transcription from the T7-SB100X plasmid (Addgene #34879) using the mMESSAGE mMACHINE kit and column purified using the MEGAclear kit (Ambion).

### CAR-T^KO^ cell production combining CRISPR and *Sleeping Beauty* transposon systems

CD4^+^ and CD8^+^ T cells were isolated from PBMCs and activated as described above. 2x10^6^ T cells were electroporated 48h after activation with 1µg of MC, 1 µg of SB100X mRNA, 3µM of each sgRNA (targeting TRAC and B2M), and 1,5µM Cas9 previously mixed as an RNP, using the ExPERT GTx™ electroporation device from MaxCyte, according to manufacturer’s instructions. CAR-T cells were expanded in TexMACS™ culture medium (Miltenyi Biotec) supplemented with 3% human serum (Sigma), 1% penicillin/streptomycin, and 625 IU/ml of human IL-7 and 85 IU/ml of human IL-15 (Miltenyi Biotec). HLA-I^KO^/TCR^KO^ CAR-T cells were negatively selected using the AutoMACS Pro Separator after incubation with anti-human HLA-I and TCRα/β antibodies and Anti-Biotin MicroBeads (Miltenyi Biotec) according to manufacturer’s instructions. For large-scale productions conditions were scaled proportionally to the number of cells.

### *Sleeping Beauty* copy number analysis

SB copy number (CN) per cell was determined by qPCR. Genomic DNA was extracted using the DNeasy Blood and Tissue Kit (Qiagen). CN/cell was quantified by duplex detection of the WPRE sequence, normalized to ALBUMIN, using specific primers, and detected with the TaqMan probes (**Table S4**). qPCR was performed using the Absolute qPCR Mix Low ROX Mix (Thermo Scientific) in a QuantStudio™ 3 Real-Time PCR System (Thermo Fisher Scientific). Results were analyzed in QuantStudio 3 Design and Analysis Software (Thermo Fisher Scientific).

### Integration site analysis

Integration site analysis was performed in genomic DNA isolated with NucleoSpin Tissue for DNA extraction kit (Macherey-Nagel) from CAR-T and CAR-T^KO^ cells (3 independent productions) using INSPIIRED pipeline (34, 35) with minor modifications as described (20). Sequencing was carried out in an Illumina MiSeq at a depth of 3-10x10^5^ reads per sample using MiSeq Reagent Kit v2 300-cycles (Illumina) (Rd1: 179c; Index1: 12c; Rd2: 143c). Raw sequencing data was demultiplexed and trimmed of ITR-specific sequences. Then, sequences were filtered against the vector sequence. The remaining unique sequences were aligned to the Human genome (GRCh38) reference using BLAT. Alignment for R1 and R2 sequences were then joined together and filtered for quality alignments, yielding unique sites of integration or multihit locations. Data were stored within an R object. Unique sites were annotated using the clusterProfiler library. The virtual machine, software, and instructions are available at https://github.com/BushmanLab/INSPIIRED.

### iGUIDE

Libraries were prepared following the protocol described in iGUIDE (36). Genomic DNA from samples was purified with NucleoSpin Tissue for DNA extraction kit (Macherey-Nagel) and randomly fragmented by ultrasonication. Adapters were ligated to end-repaired DNA, and targeted DNA was amplified through a nested-PCR from the incorporated dsODN to the ligated adapter sequence. Amplicons were purified and sequenced on an Illumina MiSeq with 300 cycle v2 reagent kits. Output sequence data was analyzed using the iGUIDE pipeline. The software and instructions are available at https://github.com/cnobles/iGUIDE.

### Statistical analysis

Statistical analyses were performed using GraphPad Prism for Mac version 10.0.0. The different tests used in this work are indicated in the figure legend.

## Results

### Design and selection of an optimized CAR targeting CD33

A panel of 12 different 4-1BB second-generation CAR constructs targeting CD33, derived from 2 different monoclonal antibodies (scFv from my96 and M195) and presenting hinge regions with different lengths from CD8a or IgG4 molecules were generated (**Figure S1A**). CAR design also included a truncated version of the human EGFR (hEGFRt) as a reporter gene to facilitate tracking of the CAR-T cells (37). The specific activation of the main signaling pathways (NFAT, NFkB, and AP1) after tumor recognition was measured using a triple reporter system in Jurkat cells (Jurkat-TPR) (38, 39). After transduction with lentiviral particles coding for the different CAR constructs Jurkat-TPR was cocultured with MOLM-13 cells, an AML cell line expressing CD33 established from the peripheral blood of an acute monocytic leukemia relapsed patient (40). Non-transduced cells (UTD) or transduced with a CAR targeting CD19 were used as controls. The top three CAR constructs [my96(45aa), my96(228aa), and M195(45aa)] showing the highest specificity without tonic signal were selected for further *in vitro* and *in vivo* functional analysis (**Figure S1B**). CAR-T cells with the selected CAR constructs were generated from healthy donors and functionally characterized. Transduction efficiency (CAR^+^ cells) and expansion capacity of CAR-T cells were similar between the three selected constructs. Evaluation of their *in vitro* lytic activity by regular cytotoxic assay against MOLM-13 cells showed that all three CAR-T cells effectively killed tumor cells at low E:T ratios. The *in vivo* antitumoral efficacy was evaluated in a xenograft model in NSG mice by administration of 3x10^6^ CAR-T cells/animal 4 days after intravenous administration of 5x10^4^ cells/animal MOLM-13 cells expressing luciferase. CAR-T cells derived from my96 antibody presented a statistically significant increased antitumor efficacy *in vivo* compared to M195(45) CAR-T cells. Finally, the my96(45) CAR, containing the 45aa hinge from CD8a, was selected for the rest of the studies (**Figures S1C–F**).

### CAR-T cells from AML patients are associated with decreased *in vitro* and *in vivo* functionality

**Figure 1:**
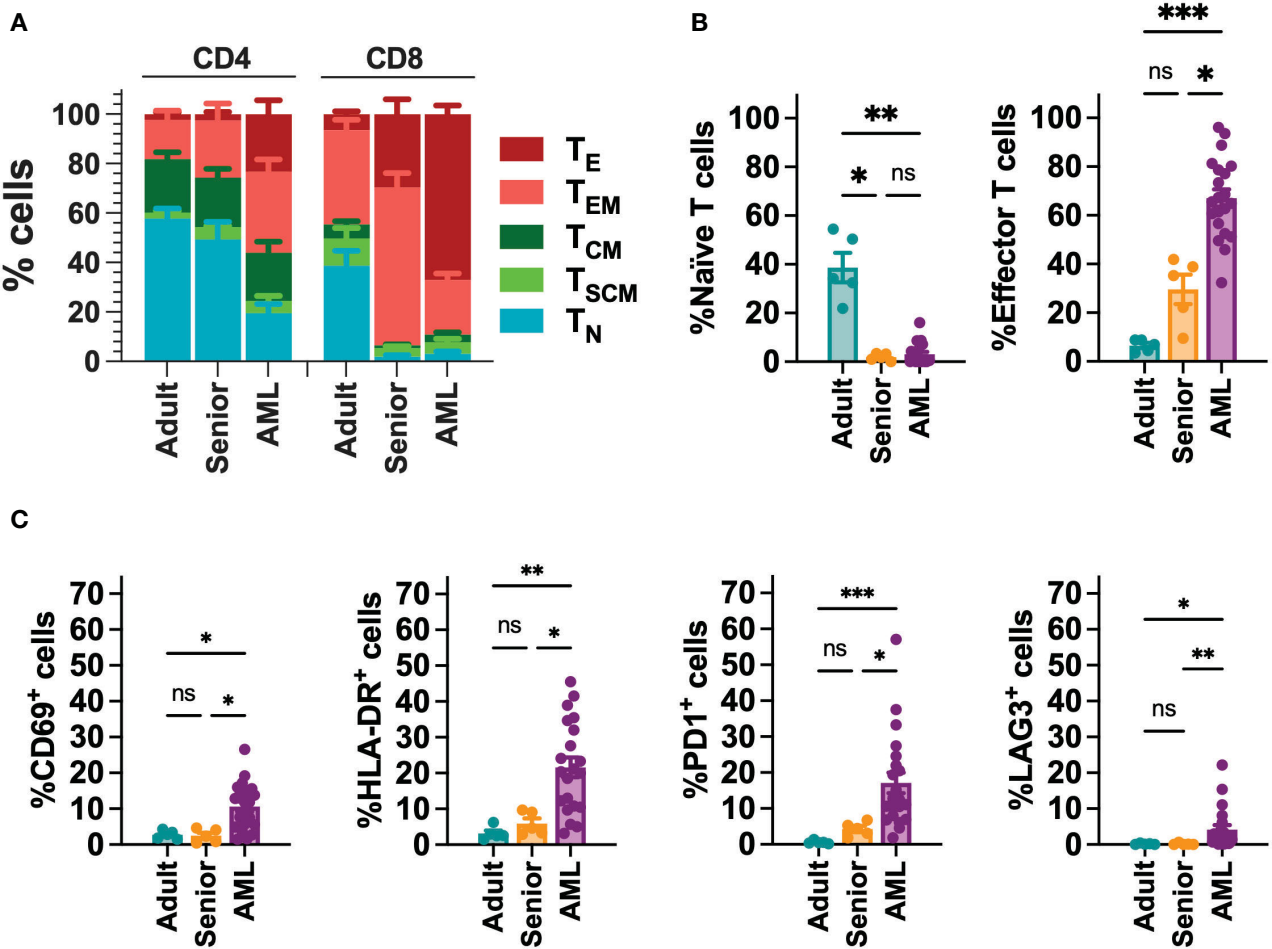
Phenotypic characterization of T cells from AML patients. **(A)** FACS analysis of T cell phenotype in AML patients (n=21), adult (below 30 years; n=5) and aged matched (senior; n=5) healthy donors. T cell subpopulations within CD4^+^ and CD8^+^ cells are depicted. T_N_: naïve; T_SCM_: stem central memory; T_CM_: central memory; T_EM_: effector memory; T_E_: effector. **(B)** Percentage of Naïve (left) and effector (right) T cell subpopulations in CD8^+^ T cells from AML patients (n=21), adult (n=5) and senior (n=5) healthy donors. **(C)** Analysis of the expression of CD69, HLA-DR, PD1 and LAG3 in CD8^+^ T cells from AML patients (n=21), adult (n=5) and senior (n=5) healthy donors. Mean ± SEM for each group is depicted. Kruskal-Wallis test with Dunn’s multiple comparisons test. ns, not significant; *p<0.05; **p<0.01; ***p<0.001.

**Figure 2:**
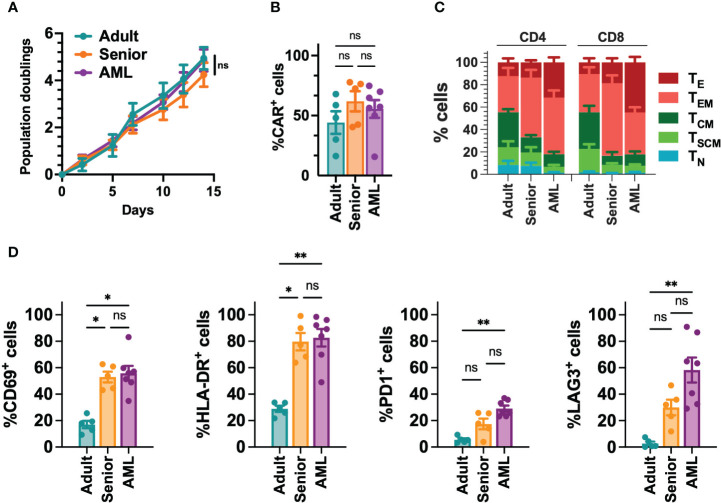
Phenotypic characterization of CD33-CAR-T cells from AML patients. **(A)** Population doublings of CAR-T cells generated from AML patients (n=7), adult (n=5) and senior (n=5) healthy donors during CAR-T cell production. **(B)** Percentage of transduced cells (CAR^+^) at the end of each CAR-T cell production. **(C)** Analysis of the phenotype of CAR-T cells at resting state for each group. CAR-T cell subpopulations within CD4^+^ and CD8^+^ cells are depicted. T_N_, naïve; T_SCM_, stem central memory; T_CM_, central memory; T_EM_, effector memory; T_E_, effector. **(D)** Analysis of the expression of CD69, HLA-DR, PD1 and LAG3 in CD8^+^ T cells from AML patients (n=7), adult (n=5) and senior (n=5) healthy donors. Mean ± SEM for each group is depicted. 2-way ANOVA with Tukey’s multiple comparisons test **(A)**, Kruskal-Wallis test with Dunn’s multiple comparisons test **(B, D)**. ns, not significant; *p<0.05; **p<0.01.

Since AML is associated with the presence of abnormal T cells phenotypes (41), we performed a phenotypic characterization of T cells collected from a cohort of 21 AML patients (**Table S5**). When compared to T cells from young adults (below 30) or aged-matched healthy donors (senior), T cells from AML patients presented a more differentiated phenotype, particularly in the CD8^+^ compartment, with significant enrichment of effector (T_E_) cells. In contrast, T cells from healthy donors were enriched in naïve T cells (T_N_) (**Figures 1A, B**, **S2**). Moreover, T cells from AML patients also displayed significantly higher expression of activation (CD69, HLA-DR) and exhaustion (PD1, LAG3) markers compared to healthy donors (**Figures 1C**, **S2**). To determine whether differences in phenotype and activation may play a role in the functionality of CAR-T cells, using our selected CAR construct, we generated CAR-T cells (CD33-CAR-T) from AML patients that were compared to CD33-CAR-T cells from young (adult) and elderly (senior) healthy donors (**Table S5**). Non-transduced T cells (UTD) from each group were used as controls. The proliferation capacity of the CD33-CAR-T and UTD cells from the different groups was similar, reaching 4-5 population doublings during the expansion phase. Moreover, similar transduction efficiency was observed between the different groups, with around 50% of CAR^+^ cells, suggesting that transduction was not impaired in AML T cells (**Figures 2A, B**). Phenotypic analysis revealed that CD33-CAR-T cells from both senior donors and AML patients presented a statistically significant reduced percentage of memory stem (T_SCM_) and central memory T cells (T_CM_), with increased levels of activation markers like CD69 and HLA-DR. However, CD33-CAR-T cells from AML patients presented an increased proportion of terminal effector (T_E_) T cells, with higher levels of PD1 and LAG3 exhaustion markers (**Figures 2C, D**, **S3**). Altogether, these data suggest that the initial T cell phenotype has a clear impact on the final phenotypic features of the CAR-T cells, with significant differences in the differentiation, activation, and exhaustion states produced by both age-related and AML-specific factors.

**Figure 3:**
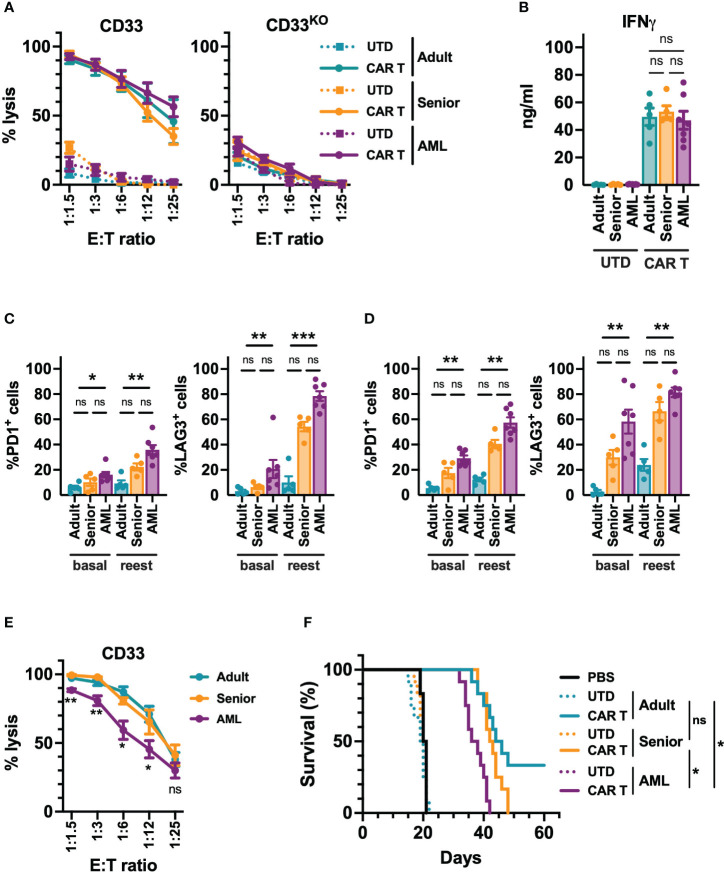
Functional characterization of CD33-CAR-T cells from AML patients. **(A)** Quantification of the cytotoxic activity of CAR-T and UTD cells generated from AML patients (n=7), adult (n=5) and senior (n=5) healthy donors, against CD33^+^ (left) and CD33 knock-out (right) MOLM-13 AML cell line at different E:T ratio. The percentage of specific lysis for each CAR-T cell production is depicted. **(B)** Quantification of IFN-γ levels in supernatants from cytotoxic assays (ratio 1:3) measured by ELISA. The cytokine concentration (ng/ml) for each CAR-T cell production is depicted. Analysis of the expression of PD1 and LAG3 in CD4^+^ **(C)** and CD8^+^ **(D)** CAR-T cells from AML patients, adult, and senior healthy donors, before (basal) and after continuous repeated *in vitro* stimulation (reest) for 21 days with MOLM-13 tumoral cells. **(E)** Cytotoxic activity of CAR-T cells from AML patients (n=7), adult (n=5) and senior (n=5) healthy donors after continuous repeated *in vitro* stimulation for 21 days with MOLM-13 tumoral cells. **(F)** Survival of mice treated with CAR-T cells from AML patients, adult, and senior healthy donors. Untreated animals or treated with UTD cell form same groups were use as control. All groups included 12 animals (6 male and 6 female). Mean ± SEM of the average of three technical replicates for each group is depicted. Kruskal-Wallis test with Dunn’s multiple comparisons test **(C, D)**, 2-way ANOVA with Tukey’s multiple comparisons test **(E)**, Logrank test **(F)**. ns, not significant; *p<0.05; **p<0.01; ***p<0.001.

Next, we decided to analyze the *in vitro* and *in vivo* functional capacity of the generated CAR-T cells. Regardless of their origin, all CD33-CAR-T cells were highly cytotoxic, producing similar levels of IFN-γ (**Figures 3A, B**, **S4**). To further explore CAR-T cell functionality under more challenging conditions, a continuous repeated *in vitro* stimulation for 21 days with tumoral cells was performed. Under these conditions, we found that CD33-CAR-T cells from AML patients presented a more exhausted phenotype, with increased PD1 and LAG3 expression, and a decreased proliferation potential that was statistically significant (**Figures 3C, D**, **S4**). These features were also associated with reduced cytotoxic capacity after repeated restimulations (**Figure 3E**). The antitumoral potential of the CAR-T cells was then evaluated *in vivo* in a xenograft model of NSG mice transplanted with MOLM-13 cells, as described above. Treatment with all CD33-CAR-T cells significantly increased animal survival compared to UTD controls with no differences between male and female animals (**Figure S4**). Interestingly, CD33-CAR-T cells from AML patients showed a statistically significant reduction of the antitumor activity with reduced survival compared to CAR-T cells from healthy donors (adult and senior) (**Figure 3F**). To address whether the decreased capacity of CD33-CAR-T from AML patients could be reverted by modifying the design on the CAR construct, we replaced 4-1BB sequence by CD28 costimulatory domain and compared CD33-CAR-T cells generated from AML patients using both constructs. Similar transduction efficiency and proliferation potential were observed between 4-1BB and CD28 CAR-T cells, with no differences in the phenotypic subpopulations, activation/exhaustion markers, cytotoxic capacity, or IFN-γ production after coculture with tumoral cells (**Figure S5**). Finally, both 4-1BB and CD28 CAR-T cells presented similar antitumoral efficacy *in vivo* in the xenograft model in NSG mice (**Figure S5**). Altogether, these data strongly suggest that the CD28 costimulatory domain does not improve the functionality of CAR-T cells from AML patients.

### Transcriptional characterization of CAR-T cells from AML patients

**Figure 4:**
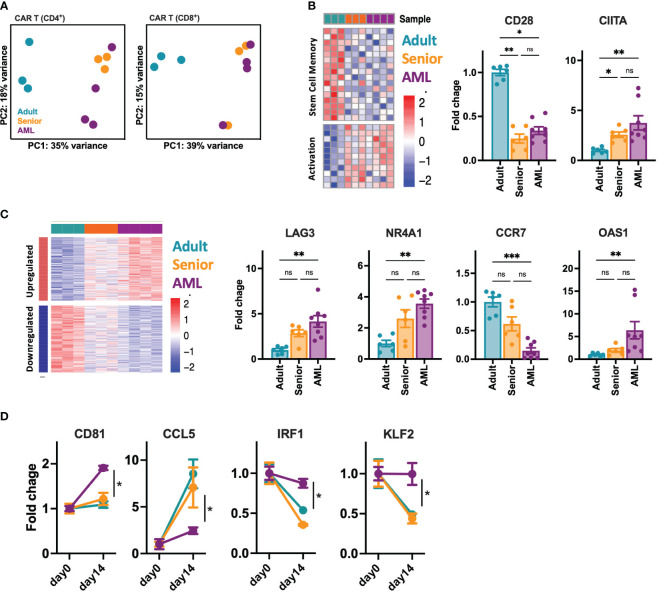
Transcriptomic characterization of CD33-CAR-T cells from AML patients. The transcriptomic landscape of CAR-T cells generated from AML patients (n=4), adult (n=3) and senior (n=3) healthy donors was profiled using high-throughput RNA sequencing (RNA-seq) **(A)** RNA-seq principal components (PC) analysis, corrected by patient heterogeneity, of sorted CD4^+^ and CD8^+^ CAR-T cell subsets. Percentage of variance explained by PC1 and PC2 are depicted. **(B)** Left: Heatmap of differentially expressed genes associated to stem cell memory and T cell activation shared between CD8^+^ CAR-T cells from AML patients and senior healthy donors (age-related) compared to adult CAR-T cells. Right: Quantification of CD28 and CIITA gene expression. **(C)** Left: Heatmap of differentially expressed genes specific for CD8^+^ CAR-T cells from AML patients (AML-specific) compared to adult and senior CAR-T cells. Right: Quantification of LAG3, NR4A1, CCR7 and OAS1 gene expression. **(D)** Quantification of CD81, CCL5, IRF1 and KLF2 gene expression as example of genes with disrupted expression pattern in AML CAR-T cells after stimulation with tumoral cells. Mean ± SEM for each group is depicted. Kruskal-Wallis test with Dunn’s multiple comparisons test **(B, C)**, 2-way ANOVA with Tukey’s multiple comparisons test **(D)**. ns, not significant; *p<0.05; **p<0.01; ***p<0.001.

Given the phenotypic and functional differences observed between CAR-T cells from AML patients and healthy donors, we further analyze CD4^+^ and CD8^+^ CD33-CAR-T cells at the transcriptomic level. RNAseq analysis before antigen recognition revealed that CAR-T cells from AML patients were transcriptionally similar to those from senior healthy donors, with only a few differentially expressed genes (DEGs) with an FDR<0.05 and a |log2FC|>1 (31 for CD4^+^ and 12 for CD8^+^ CAR-T cells) (**Table S6**). In contrast, most of the differences were observed when CAR-T cells from senior healthy donors and AML patients were compared to CAR-T cells from adult healthy donors (752 and 853 DEGs in CD4^+^ and 807 and 705 in CD8^+^ CAR-T cells respectively) (**Figure S6A**). All these differences were reflected in a principal components analysis where CAR-T cells from AML patients clustered together and close to those from senior healthy donors, being separated from CAR-T cells from adult healthy donors in both CD4^+^ and CD8^+^ CAR-T cell subsets (**Figure 4A**). A closer analysis of the DEGs revealed that most of the differences observed with adult CAR-T cells were shared between senior and AML CAR-T cells, clearly indicating age-related differences. Thus, CAR-T cells from AML patients and senior healthy donors were enriched in genes related to activation, such as CIITA, with downregulation of genes associated with stem cell memory, like CD28 (**Figure 4B**, **S6D**). Moreover, gene ontology (GO) analysis of common DEGs showed enrichment in pathways related to the regulation of lymphocyte and T cell differentiation (**Figure S6C**), which would explain the more differentiated phenotype observed in the subpopulation analysis. Interestingly, we observed that about 30-40% of the DEGs between AML and adult CAR-T cells were unique to AML condition, indicating specific differences intrinsic to AML. We observed increased expression of genes related to lymphocyte exhaustion, such as LAG3 and NR4A1. Moreover, important genes related to T cell memory (BATF3, CCR7), T cell differentiation (GATA3), apoptosis (BAX, BCL family), or IFN response (OAS1) were also deregulated (**Figure 4C**, **S6E**). In accordance, GO analysis revealed enrichment in pathways related to lymphocyte activation, response to stress and DNA damage, regulation of the immune response, antigen receptor-mediated signaling, and regulation of the cell cycle (**Figure S6C**). We further characterized CAR-T cells from AML patients upon stimulation with tumoral cells. Two weeks after stimulation we observed a set of genes presenting disrupted expression patterns (different behavior after stimulation) compared to adult and senior CAR-T cells (**Figures 4D**, **S6F**). Those genes were related to CAR-T cell migration and cell adhesion (CD81, CCL5, MMP25), proliferation (IRF1, CCR7), or regulation of cytokine production (KLF2, IL32), which would explain the reduced proliferation potential and antitumoral efficacy of these AML CAR-T cells. Altogether, these data suggest that CAR-T cells from AML patients present dysfunctional features already identified in the initial T cells, exacerbated after tumor recognition that may compromise the long-term antitumoral efficacy.

### Generation and characterization of HLA-I^KO^/TCR^KO^ CD33-CAR-T cells

**Figure 5:**
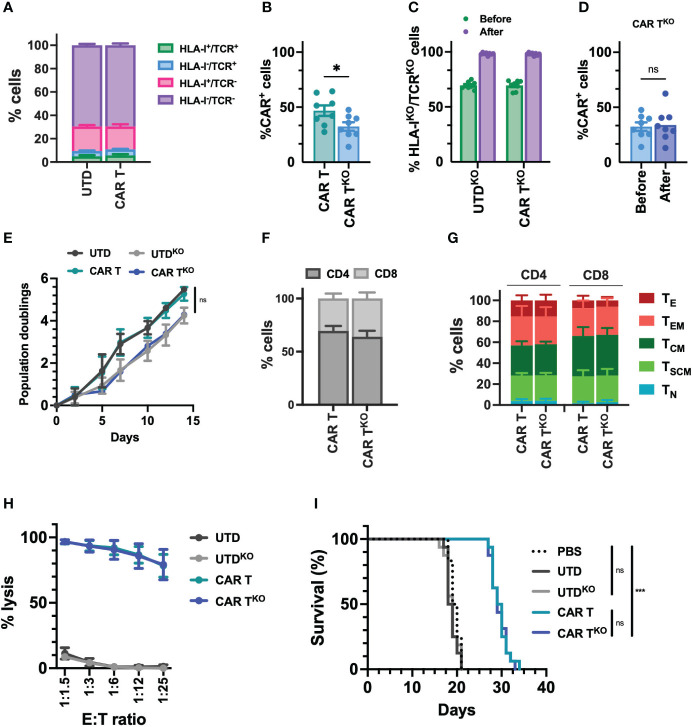
Characterization of HLA-I^KO^/TCR^KO^ CD33-CAR-T cells. Selected CRISPR RNPs were combined with the *Sleeping Beauty* transposon system to generate HLA-I^KO^/TCR^KO^ CD33-CAR-T (CAR-T^KO^) cells from healthy donors (n=8 independent samples) **(A)** Distribution of the different edited populations observed after simultaneous HLA-I and TCR targeting of CAR-T and UTD cells with CRISPR systems. **(B)** Percentage of transduced cells (CAR^+^) at the end of CAR-T and CAR-T^KO^ cell production. **(C)** Percentage of HLA-I^KO^/TCR^KO^ double negative cells in CAR-T and UTD cells before and after selection. **(D)** Percentage of transduced cells (CAR^+^) at the CAR-T^KO^ cell production before and after selection of HLA-I^KO^/TCR^KO^ double negative cells. **(E)** Population doublings of CAR-T and CAR-T^KO^ cells during CAR-T cell production. UTD and UTD^KO^ cells were use as control. **(F)** Analysis of CD4/CD8 ratio in CAR-T and CAR-T^KO^ cells. **(G)** Analysis of the phenotype of CAR-T and CAR-T^KO^ cells at resting state for each group. CAR-T cell subpopulations within CD4^+^ and CD8^+^ cells are depicted. T_N_, naïve; T_SCM_, stem central memory; T_CM_, central memory; T_EM_, effector memory; T_E_, effector. **(H)** Quantification of the cytotoxic activity of CAR-T and CAR-T^KO^ cells against CD33^+^ MOLM-13 AML cell line at different E:T ratio. The percentage of specific lysis (average of three technical replicates) for each CAR-T cell production is depicted. UTD and UTD^KO^ cells were used as control. **(I)** Survival of mice treated with CAR-T and CAR-T^KO^. Untreated animals or treated with UTD and UTD^KO^ cells were used as control. Mean ± SEM for each group is depicted. Kruskal-Wallis test with Dunn’s multiple comparisons test **(B, D)**, 2-way ANOVA with Tukey’s multiple comparisons test **(E, H)**, Logrank test **(I)**. ns, not significant; *p<0.05; ***p<0.001.

Due to the reduced potential of the CD33-CAR-T cells from AML patients and, in no less measure, the fact that delaying treatment in patients with AML may be unacceptable, we decided to approach these issues by generating allogeneic CD33-CAR-T cells. To prevent the immune rejection and graft-versus-host disease (GvHD) associated with allogeneic cells, we used CRISPR-Cas9 to knockout HLA-I and TCR expression. First, we designed different single-guide RNAs (sgRNA) targeting exon 1 of the beta-2-microglobulin (B2M) gene and exon 1 of T-cell receptor α constant (TRAC) locus, and we selected three sgRNAs based on their location and the predicted on-target/off-target efficiency (**Table S3**). Cleavage efficiency was evaluated *in vitro* by TIDE (42) after transfection of *Streptococcus pyogenes* Cas9 (SpCas9) and sgRNA ribonucleoprotein complexes (RNP) in the Jurkat cell line. β2M-sgRNA3 and TRAC-sgRNA2 were selected for further studies since they presented the highest cleavage efficacy, inducing frameshift mutations (mostly 1nt insertions) that resulted in the reduction of HLA-I and TCR levels (**Figure S7**). Then, selected CRISPR RNPs were combined with the *Sleeping Beauty* transposon system to generate HLA-I^KO^/TCR^KO^ CD33-CAR-T (CAR-T^KO^) cells from healthy donors. The transposon vector containing the selected CD33 targeting CAR construct was used as minicircle (MC) and the SB100X transposase was provided as mRNA. Our optimized protocol allowed in a single electroporation an efficient depletion of HLA-I and TCR complexes, with more than 69% of double negative cells, and an efficient CAR delivery, that was slightly reduced when CAR and RNP were delivered together (average of 46.8% vs 32.4%) (**Figures 5A, B**, **S8**). Genome-edited CD33-CAR-T cells were further purified using magnetic beads which yielded >98% double-negative cells while maintaining the percentage of transduction (CAR^+^ cells) (**Figures 5C, D**, **S8**). The proliferation capacity of CD33-CAR-T^KO^ cells, although not significant, was slightly reduced compared to non-edited CAR-T cells, an effect that was also observed in UTD^KO^ cells, consistent with the effect of electroporation (**Figure 5E**). Moreover, no differences were observed in terms of CD4/CD8 ratio, population subsets, or the expression of activation and exhaustion markers (**Figures 5F, G**, **S9**), indicating that HLA-I and TCR depletion did not affect CAR-T cell phenotype. Then *in vitro* cytotoxicity of CD33-CAR-T^KO^ cells against MOLM-13 cells was equivalent to non-edited CAR-T cells (**Figure 5H**). Moreover, although proliferation was slightly reduced after continuous restimulation with tumoral cells, CD33-CAR-T^KO^ cells also presented similar cytotoxic activity to non-edited CAR-T cells (**Figure S9**). Finally, although the *in vivo* antitumoral potential in a xenograft model in NSG mice was slightly reduced compared to previous results, where some total remissions were observed, no differences were observed between CD33-CAR-T^KO^ and CD33-CAR-T cells (**Figures 5I**, **S9**). These results indicate that CD33-CAR-T^KO^ cells generated from healthy donors could be an efficient approach to overcome dysfunctional features observed in CAR-T cells from AML patients.

### Safety analysis of HLA-I^KO^/TCR^KO^ CD33-CAR-T cells

**Figure 6:**
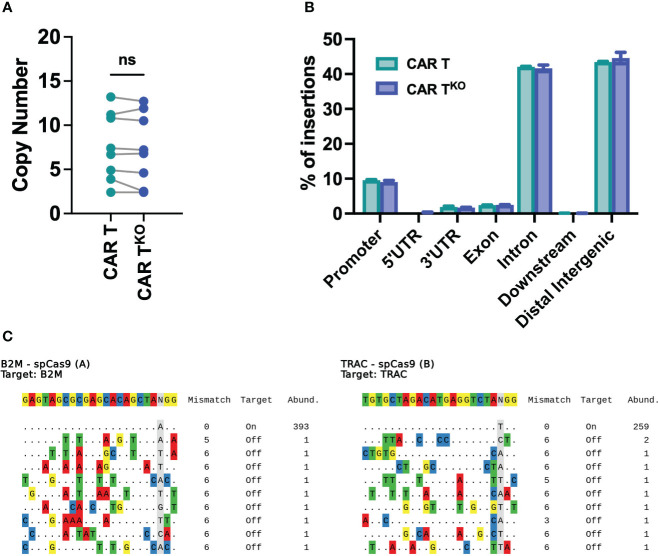
Safety analysis of HLA-I^KO^/TCR^KO^ CD33-CAR-T cells. **(A)** Analysis of the SB copy number integrations in CAR-T and CAR-T^KO^ cells (n=8 independent productions). **(B)** Histogram plot showing the genomic annotation of SB integration sites in CAR-T and CAR-T^KO^ cells (n=3 independent productions. **(C)** Sequences of cleavage sites identified by iGUIDE for B2M (left) and TRAC (right) sgRNAs annotated by on target or off target, with the total number of unique alignments associated with the site. Wilcoxon matched-pairs signed rank test **(A)**. ns, not significant.

The safety of edited cells remains a concern for human application. Analysis of genomic DNA found a similar number of transposon copies in CD33-CAR-T^KO^ cells compared to non-edited CAR-T cells generated with the *Sleeping Beauty* transposon system, with an average of 7.3 and 7.5 respectively (**Figure 6A**). Moreover, integration site analysis revealed a safe integration profile of the CAR, with no differences due to CRISPR modifications. We mapped and characterized a total of 94148 unique insertion sites of three independent CD33-CAR-T^KO^ and CAR-T cell productions. Most of the insertions were located at the expected AT-rich DNA regions of the *Sleeping Beauty* transposon system, detecting the palindromic ATATATAT motif, which contains the TA dinucleotide target sequence adjacent to all the insertions (**Figure S10**). Moreover, CAR insertions presented a wide distribution within the genome, with no preferences for promoter or exonic regions, and most of the insertions were located at distal intergenic regions (**Figures 6B**, **S10**). An additional potential issue associated with CRISPR-based genome editing would be the genotoxicity due to non-specific cleavage of the genome. Thus, we used iGUIDE (36), a modification of the GUIDE-seq method (43), to analyze the CRISPR-mediated cleavage specificity. We observed a highly specific editing of the β2M and TRAC locus, with no significant off-target sites (**Figure 6C**). Taken together, these data strongly suggest that CRISPR-mediated HLA-I and TCR knockout do not alter the safe integration profile of the *Sleeping Beauty* transposon system without inducing unspecific cleavage of the DNA.

### Preclinical production of HLA-I^KO^/TCR^KO^ CD33-CAR-T cells

**Figure 7:**
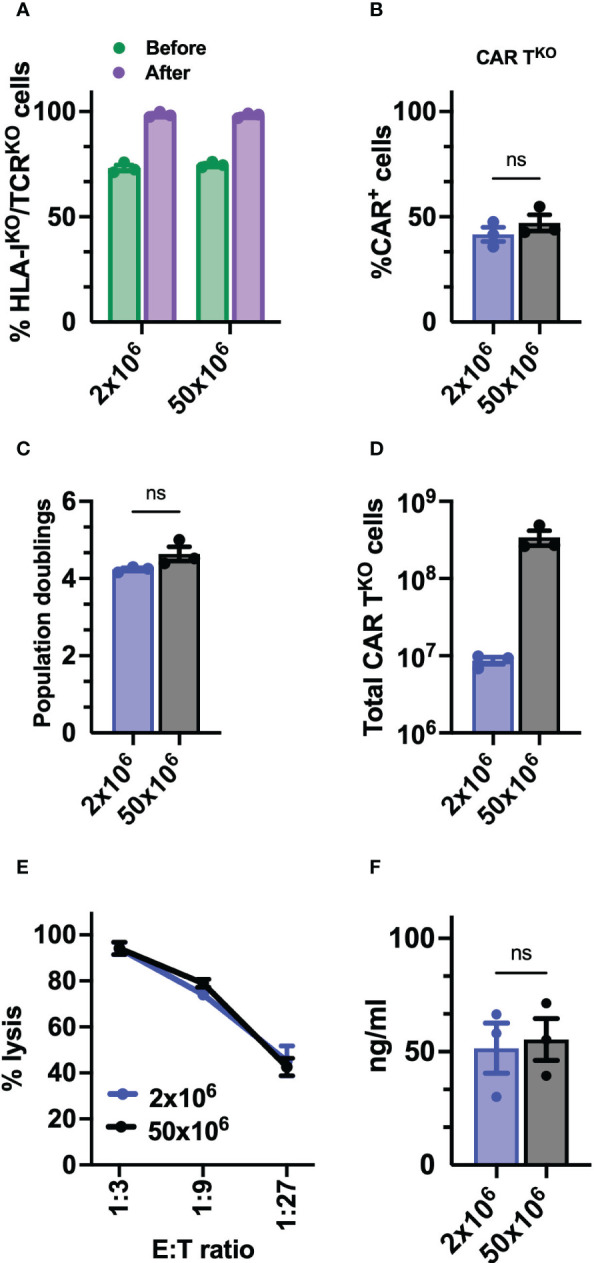
Preclinical production of HLA-I^KO^/TCR^KO^ CD33-CAR-T cells. **(A)** Percentage of HLA-I^KO^/TCR^KO^ double negative cells before and after selection (n=3 independent productions). **(B)** Percentage of transduced cells (CAR^+^) at the CAR-T^KO^ cell production after selection of HLA-I^KO^/TCR^KO^ double negative cells (n=3 independent productions). **(C)** Population doublings of CAR-T^KO^ cells during CAR-T cell production (n=3 independent productions). **(D)** Quantification of total number of CAR-T^KO^ cells obtained at the end of the production after HLA-I^KO^/TCR^KO^ double negative selection (n=3 independent productions). **(E)** Quantification of the cytotoxic activity of CAR-T^KO^ cells against CD33^+^ MOLM-13 AML cell line at different E:T ratio. The percentage of specific lysis (average of three technical replicates) for each CAR-T cell productions (n=3) is depicted. **(F)** Quantification of IFN-γ levels in supernatants from cytotoxic assays (ratio 1:3) measured by ELISA. The cytokine concentration (ng/ml; average of three technical replicates) for each CAR-T cell production (n=3) is depicted. Mann Whitney test **(B, C, F)**, 2-way ANOVA with Tukey’s multiple comparisons test **(E)**. ns, not significant.

Finally, to determine the feasibility and consistency of the process for further clinical application, we optimized the production of edited CD33-CAR-T^KO^ cells at a large scale. Thus, 50x10^6^ T cells were electroporated with equivalent scaled proportions of *Sleeping Beauty* transposon system (MC + mRNA) and RNP (Cas9 + sgRNA). Electroporated CAR-T cells were expanded using the G-Rex platform and CD33-CAR-T^KO^ cells were selected at the end of the expansion phase using AutoMACS. No differences were observed in editing efficiency before CD33-CAR-T^KO^ cell selection, with more than 70% of double negative cells, or in the purity of selected cells (**Figure 7A**). The analysis of the purified CD33-CAR-T^KO^ cells revealed no differences in the transduction efficiency and expansion capacity (population doublings), allowing the generation of at least >300x10^6^ of purified CD33-CAR-T^KO^ cells at the end of the procedure (**Figures 7B–D**). Moreover, CD33-CAR-T^KO^ cells produced at the large scale were equally functional in terms of cytotoxic activity and IFN-γ production than previously produced CD33-CAR-T^KO^ cells (**Figures 7E, F**). In summary, we have successfully developed a protocol for the efficient production and selection of fully functional gene-edited CAR-T cells, allowing the generation of large numbers of CD33-CAR-T^KO^ cells, that could be compatible with clinical applications.

## Discussion

CAR-T therapies targeting CD19 and BCMA have demonstrated impressive results in B-cell malignancies, achieving long-term responses. However, similar results have not been obtained in other hematological diseases such as acute myeloid leukemia (AML). Despite one of the main barriers hampering CAR-T cell efficacy in AML is the absence of AML-specific antigens, since most of the cell surface antigens present in AML blasts are also present in normal hematopoietic cells, for this work we selected CD33 as a model antigen for several reasons. CD33-CAR-T cells have shown some clinical efficacy (7), although mature clinical results have yet to be published, and Gemtuzumab, a monoclonal antibody directed against CD33, is already approved for the treatment of AML patients (24), supporting their role as an AML target. Moreover, allogeneic CD33-CAR-T cells have a promising therapeutic potential as a bridging therapy, prior to allogeneic hematopoietic stem cell transplantation, for some R/R AML patients. In this study, we addressed several strategies that may contribute to increasing the efficacy of CAR-T cells in AML. First have demonstrated that modifications of different CAR moieties impact the CAR-T cell response. As described for other CAR constructs (44) hinge length and scFv sequence affected CAR activation and tonic signaling, pointing out that optimal configurations are required for efficient recognition. Although this optimization cannot be generalized and should be evaluated for each antigen, we observed that CAR constructs derived from the my96 clone had better antigen-dependent responses probably due to the increased affinity for CD33 (45). The hinge length had a less critical role, but results suggested that shorter versions presented better outcomes, although more experiments should be performed to clarify this point. Other modifications such as changes in the costimulatory domain barely affected CAR-T functionality, especially *in vivo*, where similar antitumoral efficacy was observed between 4-1BB and CD28 CD33-CAR-T cells using NSG xenograft models. Differences in efficacy associated with co-stimulatory molecules have been described, in part related to activation of different signaling transduction pathways (46–48). The reasons for the absence of differences in our case might be explained in part by the aggressivity of the AML model, since a few thousand MOLM-13 cells are able to induce animal death in 2 weeks.

Although several studies have consistently indicated that the fitness of T cells is compromised in patients with AML (41), our study contributes to understanding some of the regulatory mechanisms underlying these abnormalities. Furthermore, it provides additional evidence that the fitness of the T cells has an impact on the phenotype and functional capacity of the CAR-T cells generated from those T cells. Beyond the impact that CAR-T cell manufacturing has on CAR-T cell phenotype, being more evident in senior samples, differences observed in T cells from AML patients were also translated into the CAR-T cells, presenting an even more differentiated, activated, and exhausted phenotypes, that resulted in a reduced antitumoral efficacy both *in vitro* and *in vivo*. Interestingly, we identified changes that were age-related, since they were also observed in CAR-T cells from aged-matched healthy donors, which were more exacerbated after tumor recognition in AML CAR-T cells. Other differences, however, were intrinsic to AML, in particular the higher proportion of terminally differentiated effector CD8^+^ CAR-T cells with increased levels of exhaustion markers. These results are in accordance with studies performed in CAR-T cells from MM patients (49), another hematological disease mostly developed in elderly people. These features would explain the differences observed in the functionality of those AML CAR-T cells and would suggest that autologous CAR-T cell approaches could not be the best option for AML treatment. Recent studies have proposed the use of matched donor-derived allogeneic cells, that could partially overcome some of the limitations of autologous CAR-T cells (9, 10).

The use of allogeneic cells for the generation of CAR-T cells has been proposed as a means to generate an off-the-shelf product which has a number of advantages over autologous products. The use of fitter T-cells, the potential to generate many different dosages from a single donor and with significant reduction in costs need to be balanced with the limitations associated with allogeneic cells such as the development of GVHD and the need for additional immunosuppression to facilitate engraftment (9). Recent clinical studies have demonstrated the possibility of manufacturing gene-edited CAR-T cells for the treatment of patients with MM or ALL (50, 51). Different technologies, including TALEN nucleases or CRISPR systems, have been explored to prevent immune rejection and GvHD, generally by eliminating the expression of endogenous TCR and HLA-I complexes. Editing of additional genes (i.e. PD1, CD52, HLA-II, etc…) would represent a promising option to improve allogeneic CAR-T therapies, by increasing antitumoral efficacy (PD1 disruption) and/or increasing CAR-T cell persistence (CD52 knock-out) (9, 13). Moreover, the use of novel gene editing technologies, such as base-editing tools, would represent a promising and safer option under evaluation for the generation of allogeneic CAR-T cells (52). However, the large-scale production compatible with clinical applications, as well as the clinical efficacy and safety of these multiple-gene edited CAR-T cells still needs to be demonstrated. Our results have combined two state-of-the-art technologies to generate a ready-to-use allogeneic CD33-CAR-T product, the CRISPR systems and non-viral *Sleeping Beauty* (SB) based transposon systems. We have optimized the manufacturing protocol reducing the critical steps that compromise cell viability and simplifying the overall procedure. In contrast to recently published protocols (18, 53), where the CAR transduction and editing steps are performed sequentially, our protocol uses a single electroporation step for the efficient delivery of the CAR and the efficient depletion of both HLA-I and TCR complexes. Although the transduction efficiency is slightly reduced in the edited cell, with a lower number of CAR^+^ cells our protocol is able to generate enough cells compatible with clinical applications. In accordance with previous studies (18, 53), we have also demonstrated that double HLA-I and TCR-depleted CAR-T cells (CAR-T^KO^ cells) have similar features to non-edited CAR-T cells. Moreover, although some variabilities were observed in the *in vivo* antitumoral efficacy, probably due to the aggressivity of the tumor model and the donor’s variability, CAR-T^KO^ cells are fully functional. In summary, although additional studies related to CAR-T^KO^ cell persistence could be performed to better characterize this aspect in long-term applications, our results would support the use of these CAR-T^KO^ cells for therapeutic approaches in R/R AML patients where CAR-T cells are used to reduce tumor burden as bridging therapy prior to allogeneic stem cell transplantation.

We have additionally performed a set of analyses to confirm the safety of CAR-T^KO^ cells. The combination of the SB transposon system with CRISPR ribonucleoprotein (RNP) does not alter either the vector copy number or the insertion site, indicating that CAR-T^KO^ cells maintain the safe integration profile described for SB transposon system (21, 22, 54). Moreover, off-target analysis using improved technologies for unbiased genome-wide analysis, corroborated that CRISPR-mediated modifications were specific without significant off-targets. Although the absence of off-targets was expected for the sgRNA targeting the TRAC locus, since it was already described and validated in the literature (55), the b2M sgRNA was specifically designed for this study, and safety assessment was a requirement for potential clinical translation. Our results clearly indicate that the combination of these two sgRNAs are very efficient and specific. Moreover, based on previous results using the same or similar sgRNAs (56, 57), we could anticipate that safety issues related to chromosomal rearrangements would be unlikely. In the future, and in order to move to clinical application additional analysis, like CAST-seq, should be performed to corroborate genomic safety. Finally, we have demonstrated that our protocol is scalable, being able to produce sufficient CAR-T^KO^ cells compatible with clinical applications without compromising CAR-T functionality and purity. Moreover, our protocol for the generation of CAR-T^KO^ cells is not restricted to CD33-targeting CAR-T cells, since delivery of the CAR construct and the HLA-I and TCR knock-out processes, although performed together, are independent processes. Thus, HLA-I and TCR disruption (or even other genes of interest) could be combined with any other CAR construct targeting a different antigen, either for AML treatment (i.e. CLL1 or CD44v6) or another relevant disease (i.e. solid tumors). Although currently CAR-T cell manufacturing has a tendency toward the automatization of procedures, as described recently (53), our approach reduces the number of steps required for production. Moreover, the expansion of the cells in G-Rex devices is compatible with further automatizations in the fill and finish steps, which should be optimized using GMP procedures.

In summary, our work demonstrates that CAR-T cells from AML patients, although functional, present phenotypic and functional features that could compromise their antitumoral efficacy, compared to CAR-T cells from healthy donors. The combination of CRISPR technologies with transposon-based delivery strategies allows the generation of HLA-I^KO^/TCR^KO^ CAR-T cells, compatible with allogeneic approaches, that would represent a promising option for AML treatment.

## Data availability statement

The original contributions presented in the study are publicly available. This data can be found here: https://www.ncbi.nlm.nih.gov/geo/query/acc.cgi?acc=GSE239960.

## Ethics statement

The studies involving humans were approved by Research Ethics Committee of the University of Navarra. The studies were conducted in accordance with the local legislation and institutional requirements. The participants provided their written informed consent to participate in this study. The animal study was approved by Ethics Committee of the University of Navarra. The study was conducted in accordance with the local legislation and institutional requirements.

## Author contributions

CCa: Formal Analysis, Investigation, Writing – original draft. CCe: Formal Analysis, Investigation, Writing – original draft. AA: Conceptualization, Writing – original draft. PJ: Investigation, Writing – review & editing. MC: Formal Analysis, Software, Writing – review & editing. PM: Investigation, Writing – review & editing. PR: Investigation, Writing – original draft. AM: Investigation, Writing – review & editing. EI: Investigation, Writing – review & editing. GA: Investigation, Writing – review & editing. SR: Investigation, Writing – review & editing. RM: Investigation, Writing – review & editing. JI: Investigation, Resources, Writing – review & editing. MV: Resources, Writing – review & editing. MR: Resources, Writing – review & editing. JR: Resources, Writing – review & editing. SV: Resources, Writing – review & editing. JL: Conceptualization, Writing – review & editing. SI: Conceptualization, Investigation, Writing – review & editing. Ad: Conceptualization, Investigation, Writing – review & editing. MH: Conceptualization, Formal Analysis, Funding acquisition, Software, Writing – original draft, Writing – review & editing. FP: Conceptualization, Funding acquisition, Writing – original draft, Writing – review & editing. JR: Conceptualization, Writing – original draft, Writing – review & editing, Formal Analysis, Funding acquisition.

## Supplementary material

Supplementary Table 1 List of the antibodies used in this study.

Supplementary Table 2 List of the gene signatures.

Supplementary Table 3 List of the sgRNAs sequences and scores.

Supplementary Table 4 List of the primer and probe sequences used in this study.

Supplementary Table 5 Clinical characteristics of AML patients.

Supplementary Table 6 List of differentially expressed genes in RNA-seq analysis.
