# Supplementary material for: Optimization of universal allogeneic CAR-T cells combining CRISPR and transposon-based technologies for treatment of acute myeloid leukemia
Source: Front Immunol. 2023 Sep 19;14:1270843. doi: 10.3389/fimmu.2023.1270843 (PMC10546312; doi:10.3389/fimmu.2023.1270843)
Supplement: Supplementary file 1 [file DataSheet_1.pdf]

Supplementary Figures

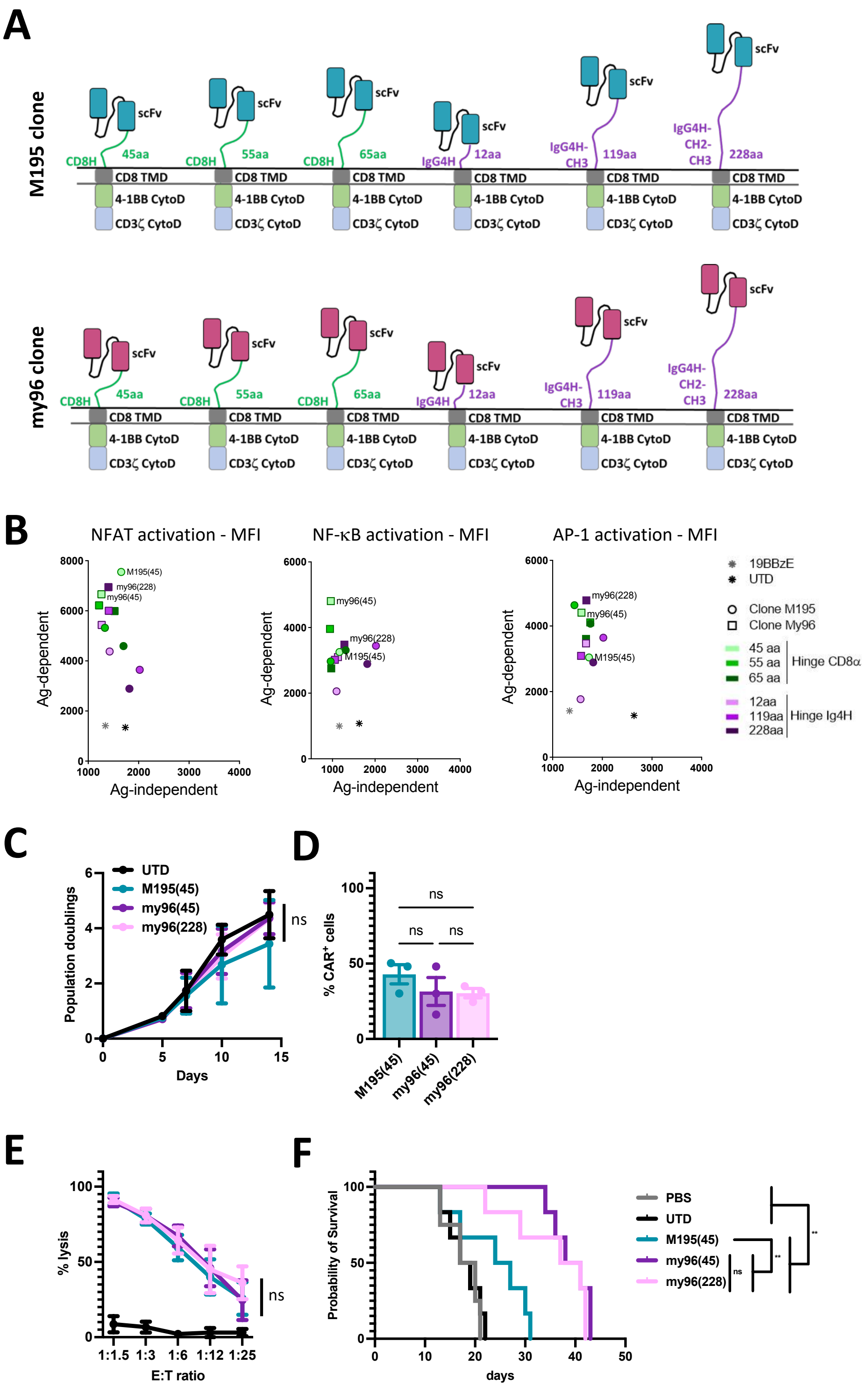

Fig. S1. Phenotypic characterization of T cells from AML patients. (A) Schematic representation of the different 4-1BB second-generation CAR constructs targeting CD33 derived from 2 different monoclonal antibodies (scFv from my96 and M195) and presenting hinge regions with different

lengths from CD8a or IgG4 molecules. **(B)** Measurement of the mean fluorescence intensity (MFI) of the NFAT (GFP; left panel), NF-κB (CFP; middle panel) and AP-1 (mCherry; right panel) pathways 24h after co-culture of Jurkat TPR cells infected with the different CAR constructs with MOLM-13 tumoral cells. Non-infected Jurkat TPR cells and expressing a CD19-targeting CAR were used as control. Average of 5 independent experiments is shown. **(C)** Population doublings of indicated CAR-T cells during CAR-T cell production (n=3 independent productions). **(D)** Percentage of transduced cells (CAR^+) at the end of each CAR-T cell production (n=3 independent productions). **(E)** Quantification of the cytotoxic activity of indicated CAR-T cells against $CD33^+$ MOLM-13 AML cell line at different E:T ratio. The percentage of specific lysis for each CAR-T cell production (n=3) is depicted. **(F)** Survival of mice treated with indicated CAR-T cells. Untreated animals or treated with UTD cell form same groups were used as control. Mean ± SEM for each group is depicted. 2-way ANOVA with Tukey's multiple comparisons test (C and E), Kruskal-Wallis test with Dunn's multiple comparisons test (D), Logrank test (F). ns: not significant; *$p<0.05$; **$p<0.01$.
