# Supplementary material for: Optimization of universal allogeneic CAR-T cells combining CRISPR and transposon-based technologies for treatment of acute myeloid leukemia
Source: Front Immunol. 2023 Sep 19;14:1270843. doi: 10.3389/fimmu.2023.1270843 (PMC10546312; doi:10.3389/fimmu.2023.1270843)
Supplement: Supplementary file 2 [file DataSheet_2.pdf]

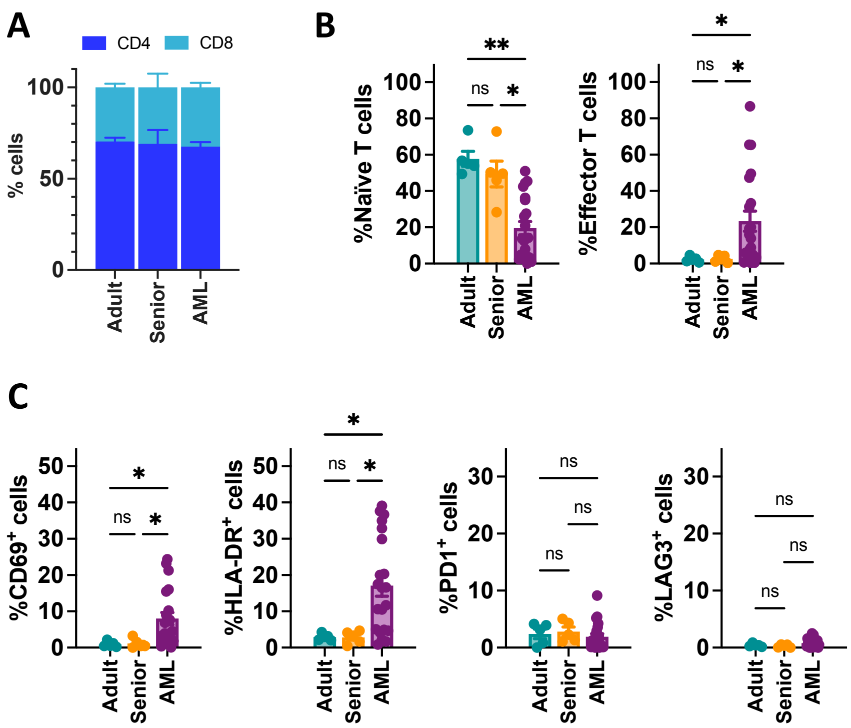

Fig. S2. Phenotypic characterization of T cells from AML patients. (A) Analysis of CD4/CD8 ratio in T cells from AML patients (n=21), adult (n=5), and senior (n=5) healthy donors. **(B)** Percentage of Naïve (left) and effector (right) T cell subpopulations in $CD4^+$ T cells from AML patients (n=21), adult (n=5), and senior (n=5) healthy donors . **(C)** Analysis of the expression of CD69, HLA-DR, PD1 and LAG3 in $CD4^+$ T cells from AML patients (n=21), adult (n=5), and senior (n=5) healthy donors . Mean ± SEM for each group is depicted. Kruskal-Wallis test with Dunn's multiple comparisons test. ns: not significant; *p<0.05; **p<0.01.
