# Supplementary material for: Optimization of universal allogeneic CAR-T cells combining CRISPR and transposon-based technologies for treatment of acute myeloid leukemia
Source: Front Immunol. 2023 Sep 19;14:1270843. doi: 10.3389/fimmu.2023.1270843 (PMC10546312; doi:10.3389/fimmu.2023.1270843)
Supplement: Supplementary file 3 [file DataSheet_3.pdf]

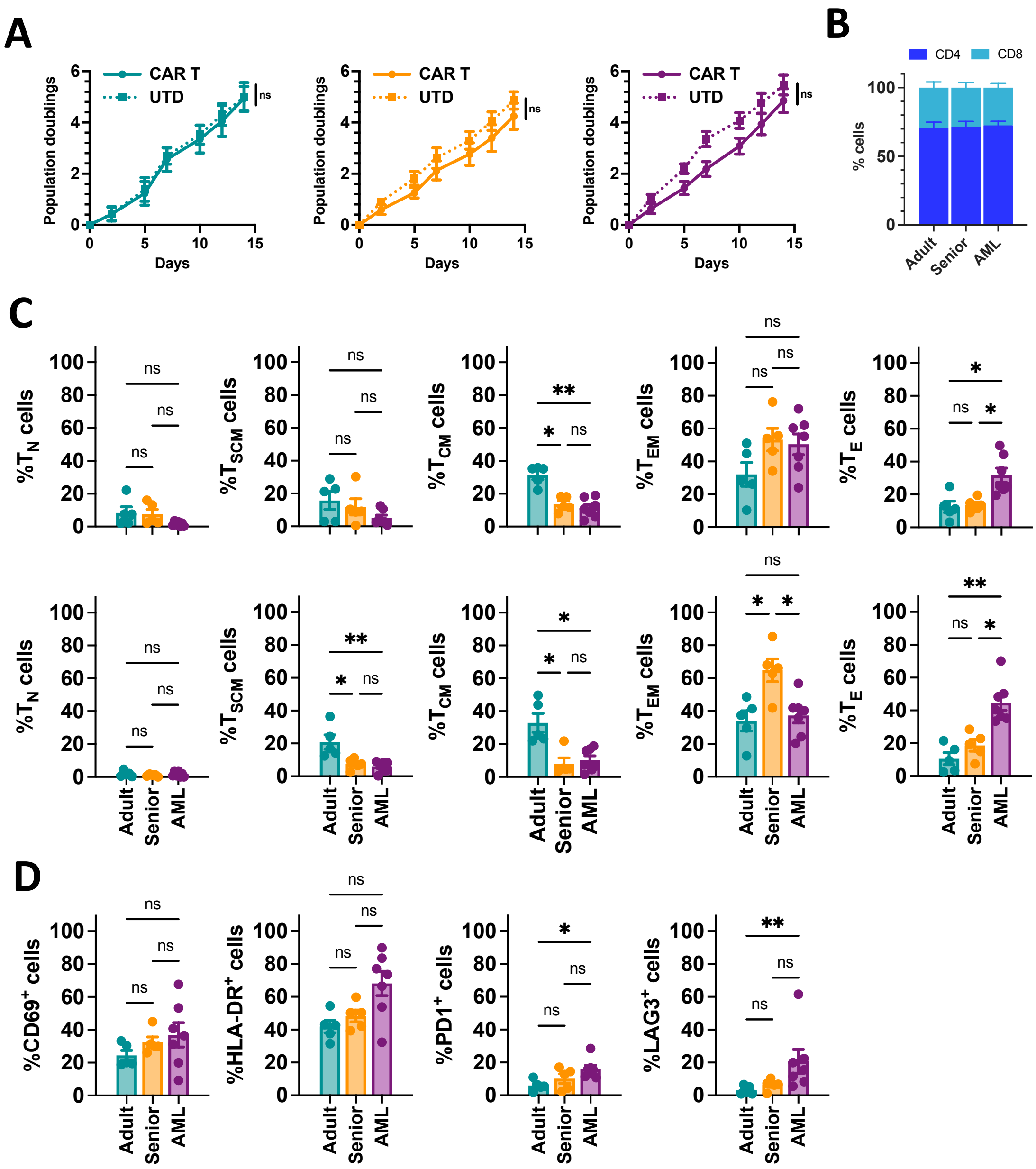

Fig. S3. Characterization of CD33-CAR-T cells from AML patients. (A) Population doublings of CAR-T cells generated from AML patients (n=7), adult (n=5), and senior (n=5) healthy donors during CAR-T cell production. **(B)** Analysis of CD4/CD8 ratio in CAR-T cells from AML patients (n=7), adult (n=5), and senior (n=5) healthy donors . **(C)** Analysis of the phenotype of CAR-T cells at resting state for each group. CAR-T cell subpopulations within $CD4^+$ (upper panel) and $CD8^+$ cells (lower panel) are depicted. T_N: naïve; T_{SCM}: stem central memory; T_{CM}: central memory; T_{EM}: effector memory; T_E: effector. **(D)** Analysis of the expression of CD69, HLA-DR, PD1 and LAG3 in $CD4^+$ CAR-T cells from AML patients (n=7), adult (n=5), and senior (n=5) healthy donors . Mean ± SEM for each group is depicted. 2-way ANOVA with Tukey's multiple comparisons test (A), Kruskal-Wallis test with Dunn's multiple comparisons test (C and D). ns: not significant; *p<0.05; **p<0.01.
