# Supplementary material for: Optimization of universal allogeneic CAR-T cells combining CRISPR and transposon-based technologies for treatment of acute myeloid leukemia
Source: Front Immunol. 2023 Sep 19;14:1270843. doi: 10.3389/fimmu.2023.1270843 (PMC10546312; doi:10.3389/fimmu.2023.1270843)
Supplement: Supplementary file 4 [file DataSheet_4.pdf]

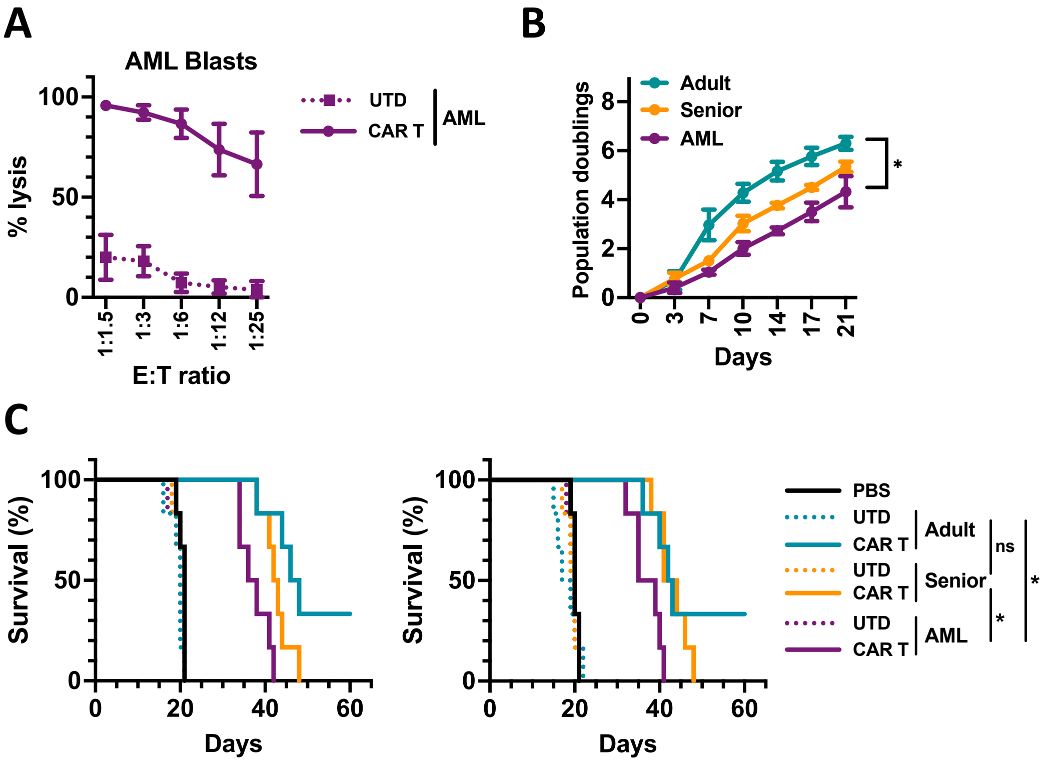

Fig. S4. Functional characterization of CD33-CAR-T cells from AML patients. (A) Quantification of the cytotoxic activity of AML CAR-T cells against primary AML blasts at different E:T ratio. The percentage of specific lysis (average of three technical replicates) for each CAR-T cell production (n=3) is depicted. **(B)** Population doublings of indicated CAR-T cells after continuous repeated *in vitro* stimulation for 21 days with MOLM-13 tumoral cells. **(C)** Survival of mice treated with indicated CAR-T cells. Untreated animals or treated with UTD cell from same groups were used as control. All groups included 12 animals (6 male and 6 female). Mean ± SEM for each group is depicted. 2-way ANOVA with Tukey's multiple comparisons test (B), Logrank test (C). ns: not significant; *p<0.05.
