# Supplementary material for: Optimization of universal allogeneic CAR-T cells combining CRISPR and transposon-based technologies for treatment of acute myeloid leukemia
Source: Front Immunol. 2023 Sep 19;14:1270843. doi: 10.3389/fimmu.2023.1270843 (PMC10546312; doi:10.3389/fimmu.2023.1270843)
Supplement: Supplementary file 5 [file DataSheet_5.pdf]

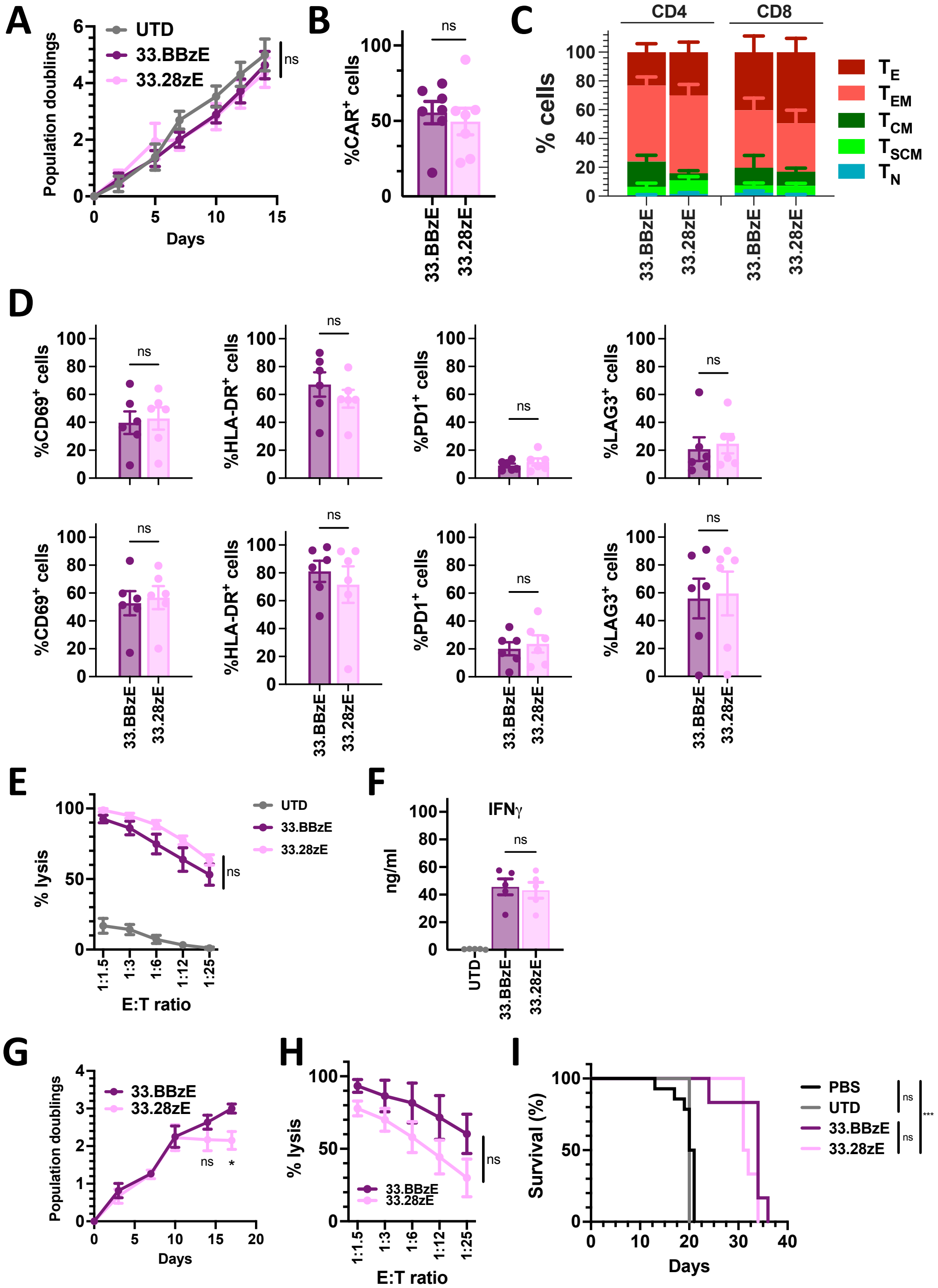

Fig. S5. Comparison of CD33-CAR-T cells from AML patients with 4-1BB or CD28 domains. (A) Population doublings of indicated CAR-T cells during CAR-T cell production (n=6-7/group). **(B)** Percentage of transduced cells (CAR^+) at the end of each CAR-T cell production (n=6-7/group). **(C)** Analysis of the phenotype of CAR-T cells at resting state for each group (n=6-7/group). CAR-T cell subpopulations within $CD4^+$ and $CD8^+$ cells are depicted. T_N: naïve; T_{SCM}: stem central memory; T_{CM}: central memory; T_{EM}: effector memory; T_E: effector. **(D)** Analysis of the expression of CD69, HLA-DR, PD1 and LAG3 in $CD4^+$ (upper panel) and $CD8^+$ (lower panel) CAR-T cells from AML patients with 4-1BB or CD28 co-stimulatory domain (n=6-7/group). **(E)** Quantification of the cytotoxic activity

of indicated CAR-T cells against $CD33^+$ MOLM-13 AML cell line at different E:T ratio. The percentage of lysis (average of three technical replicates) for each CAR-T cell production (n=6-7/group) is depicted. **(F)** Quantification of IFNγ levels in supernatants from cytotoxic assays (ratio 1:3) measured by ELISA. The cytokine concentration (ng/ml; average of three technical replicates) for each CAR-T cell production is depicted (n=6-7/group). **(G)** Population doublings of indicated CAR-T cells during repeated stimulation with MOLM-13 AML cell line. **(H)** Quantification of the cytotoxic activity of CAR-T cells against $CD33^+$ MOLM-13 AML cell line at different E:T ratio after repeated stimulation with MOLM-13 AML cell line. The percentage of lysis (average of three technical replicates) for each CAR-T cell production (n=3) is depicted. **(I)** Survival of mice treated with indicated CAR-T cells (n=6 per group). Untreated animals (PBS; n=13)) or treated with UTD (n=6) cell from same groups were used as control. Mean ± SEM for each group is depicted. 2-way ANOVA with Tukey's multiple comparisons test (A, E, G and H), Mann Whitney test (B and D), Kruskal-Wallis test with Dunn's multiple comparisons test (F), Logrank test (I). ns: not significant; *p<0.05; **p<0.01, ***p<0.001.
