# Supplementary material for: Optimization of universal allogeneic CAR-T cells combining CRISPR and transposon-based technologies for treatment of acute myeloid leukemia
Source: Front Immunol. 2023 Sep 19;14:1270843. doi: 10.3389/fimmu.2023.1270843 (PMC10546312; doi:10.3389/fimmu.2023.1270843)
Supplement: Supplementary file 6 [file DataSheet_6.pdf]

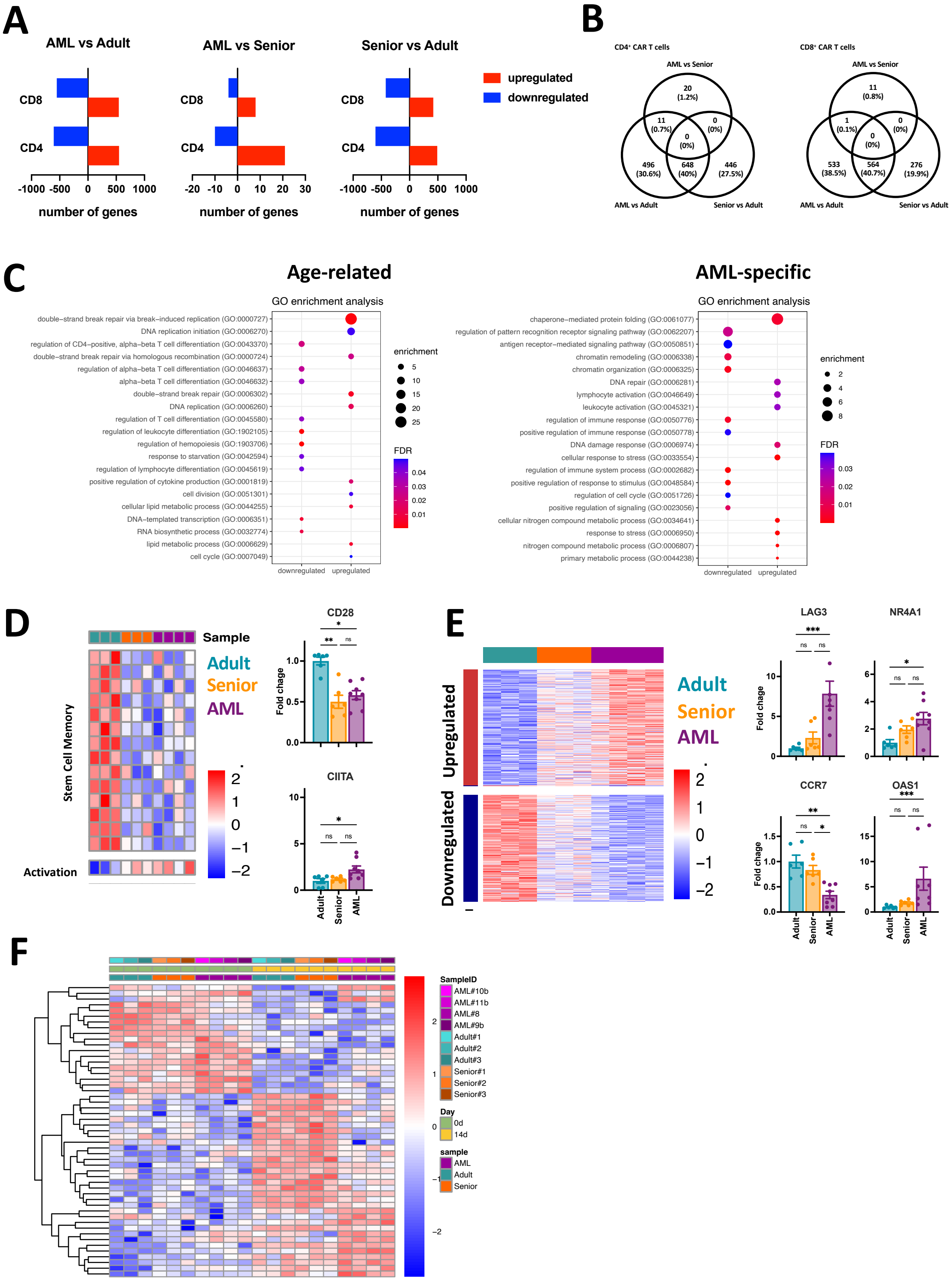

Fig. S6. Transcriptomic characterization of CD33-CAR-T cells from AML patients. The transcriptomic landscape of CAR-T cells generated from AML patients (n=4), adult (n=3) and senior (n=3) healthy donors was profiled using high-throughput RNA sequencing (RNA-seq). **(A)** Number of differentially expressed genes (DEGs) with an FDR<0.05 and a |Log2FC|>1 between indicated groups.

(B) Venn's diagram showing DEGs between all groups. **(C)** Age-related and AML-specific gene ontology (GO) enrichment analysis. **(D)** Heatmap and quantification of differentially expressed genes (DEGs) between $CD4^+$ CAR-T cells from AML patients, adult and senior healthy donors associated to genes involved in stem cell memory and T cell activation. **(E)** Heatmap and quantification of AML-specific DEGs in $CD4^+$ CAR-T cells from AML patients, compared to adult and senior healthy donors. **(F)** Heatmap of genes with disrupted expression pattern after antigen stimulation in AML CAR-T cells compared to adult and senior healthy donors. Mean ± SEM for each group is depicted. Kruskal-Wallis test with Dunn's multiple comparisons test (D and E). ns: not significant; *p<0.05; **p<0.01; ***p<0.001.
