# Supplementary material for: Optimization of universal allogeneic CAR-T cells combining CRISPR and transposon-based technologies for treatment of acute myeloid leukemia
Source: Front Immunol. 2023 Sep 19;14:1270843. doi: 10.3389/fimmu.2023.1270843 (PMC10546312; doi:10.3389/fimmu.2023.1270843)
Supplement: Supplementary file 7 [file DataSheet_7.pdf]

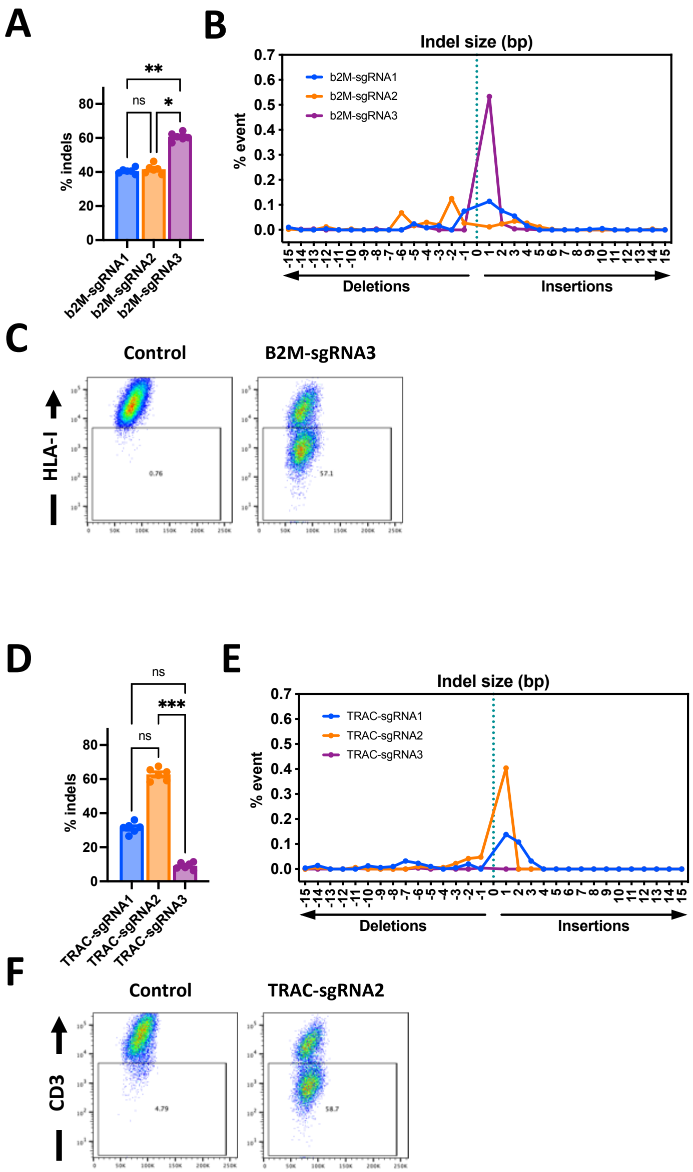

Fig. S7. Characterization of sgRNAs targeting B2M and TRAC locus. (A) Cleavage efficiency of B2M sgRNAs measured by TIDE in Jurkat cell. Percentage of indels of 6 independent experiments is depicted. **(B)** Percentage and size of indels of B2M sgRNAs around the target site (average of 6 independent experiments). **(C)** Analysis of the expression of HLA-I in Jurkat cells after 72h after B2M-sgRNA3:RNP electroporation (a representative experiment is shown). **(D)** Cleavage efficiency of TRAC sgRNAs measured by TIDE in Jurkat cell. Percentage of indels of 6 independent experiments is depicted . **(E)** Percentage and size of indels of TRAC sgRNAs around the target site (average of 6 independent experiments). **(C)** Analysis of the expression of CD3 in Jurkat cells after 72h after TRAC-sgRNA2:Cas9 RNP electroporation (a representative experiment is shown).
