# Supplementary material for: Optimization of universal allogeneic CAR-T cells combining CRISPR and transposon-based technologies for treatment of acute myeloid leukemia
Source: Front Immunol. 2023 Sep 19;14:1270843. doi: 10.3389/fimmu.2023.1270843 (PMC10546312; doi:10.3389/fimmu.2023.1270843)
Supplement: Supplementary file 8 [file DataSheet_8.pdf]

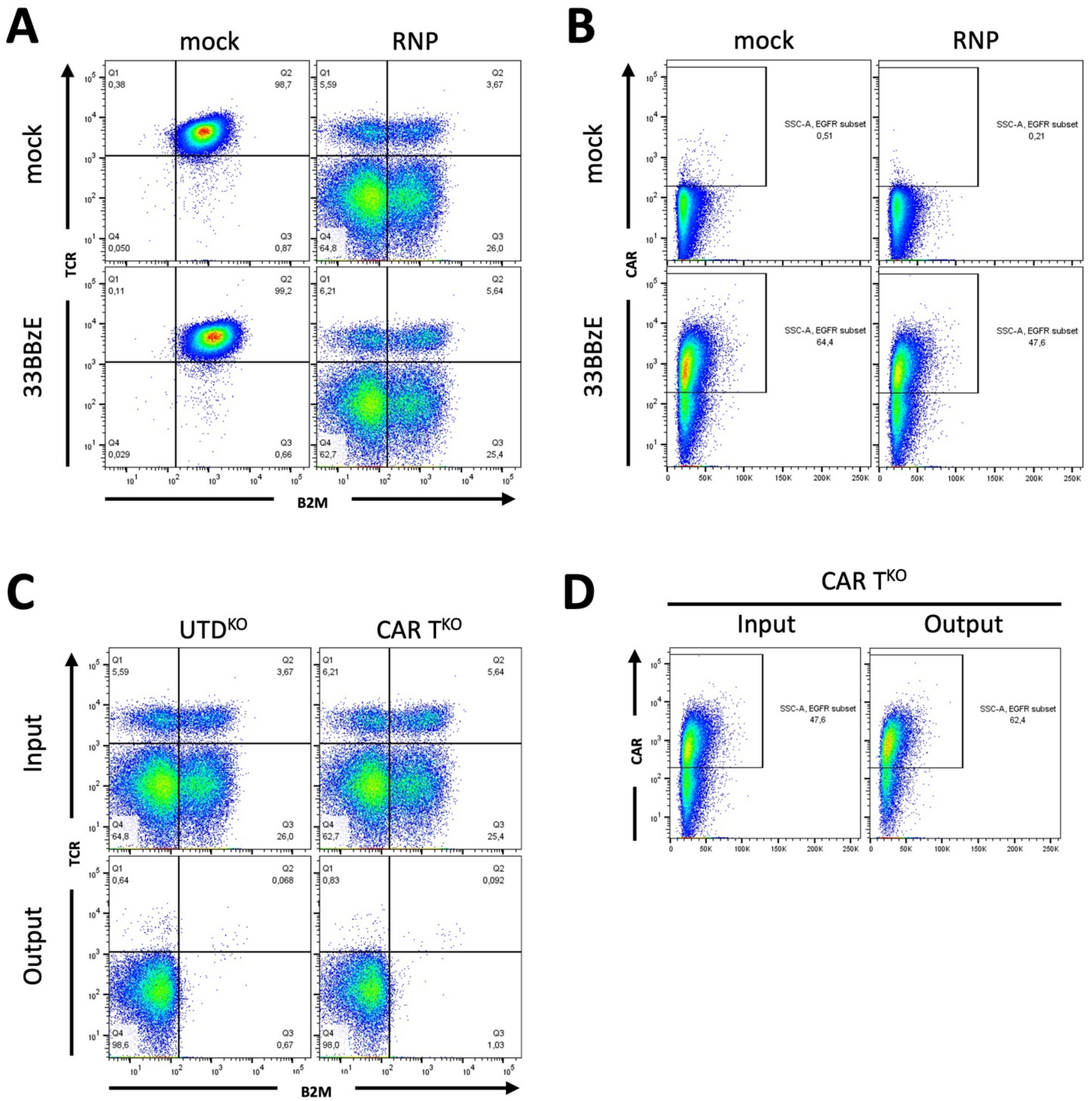

Fig. S8. Phenotypic characterization of HLA-I^{KO}/TCR^{KO} CD33-CAR-T cells. Representative plots of the analysis of TCR and B2M **(A)** and CAR **(B)** expression 14 days after electroporation of T cells with selected CRISPR RNPs (targeting TRAC and B2M) combined with the *Sleeping Beauty* transposon system expressing the selected CAR targeting CD33. T cells electroporated only with CRISPR RNPs or *Sleeping Beauty* transposon system, and non-electroporated T cells were used as control. Representative plots of the analysis of TCR and B2M **(C)** and CAR **(D)** expression in genome-edited CD33-CAR-T cells before (input) and after (output) purification with magnetic beads.
