# Supplementary material for: Optimization of universal allogeneic CAR-T cells combining CRISPR and transposon-based technologies for treatment of acute myeloid leukemia
Source: Front Immunol. 2023 Sep 19;14:1270843. doi: 10.3389/fimmu.2023.1270843 (PMC10546312; doi:10.3389/fimmu.2023.1270843)
Supplement: Supplementary file 9 [file DataSheet_9.pdf]

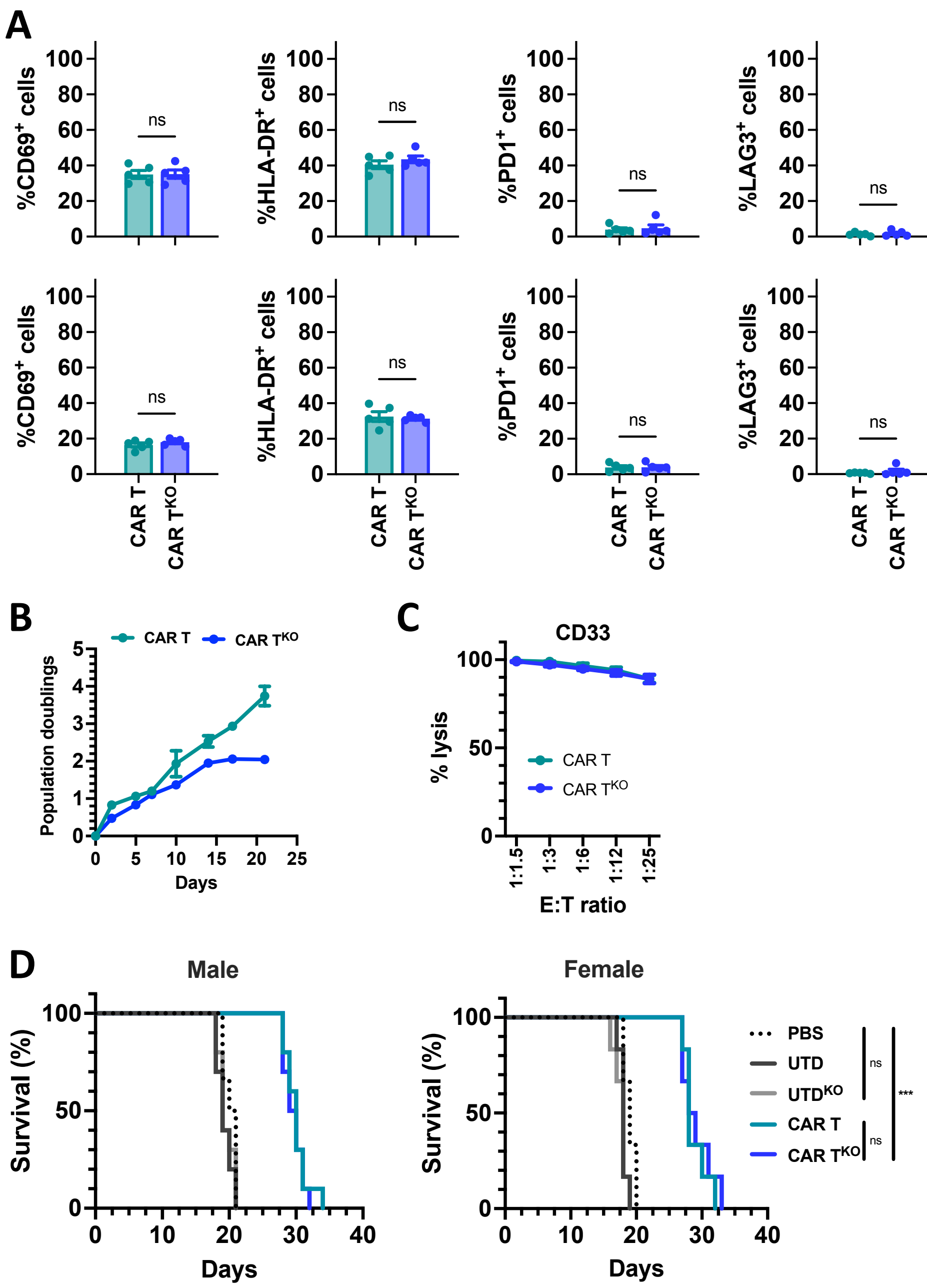

Fig. S9. Phenotypic and *in vivo* characterization of HLA-I^{KO}/TCR^{KO} CD33-CAR-T cells. (A) Analysis of CD69, HLA-DR, PD1 and LAG3 expression in $CD4^+$ (upper panel) and $CD8^+$ (lower panel) HLA-I^{KO}/TCR^{KO} CD33-CAR-T cells (CAR-T^{KO}; n=5 independent productions). Non-edited CAR-T cells were used as control (n=5 independent prorductions). **(B)** Population doublings of indicated CAR-T cells during repeated stimulation with MOLM-13 AML cell line. **(C)** Quantification of the cytotoxic activity of CAR-T cells against $CD33^+$ MOLM-13 AML cell line at different E:T ratio after repeated stimulation with MOLM-13 AML cell line. The percentage of lysis (average of three technical replicates) for each CAR-T cell production (n=3) is depicted. **(D)** Survival of mice treated with indicated CAR-T cells (n=16, 10 male and 6 female). Untreated animals (PBS; n=12, 6 male and

6 female) or treated with UTD cells from same groups (n=16, 10 male and 6 female) were used as control. Mean ± SEM for each group is depicted. Mann Whitney test (A), Logrank test (B). ns: not significant; ***$p<0.001$.
