# Supplementary material for: Optimization of universal allogeneic CAR-T cells combining CRISPR and transposon-based technologies for treatment of acute myeloid leukemia
Source: Front Immunol. 2023 Sep 19;14:1270843. doi: 10.3389/fimmu.2023.1270843 (PMC10546312; doi:10.3389/fimmu.2023.1270843)
Supplement: Supplementary file 10 [file DataSheet_10.pdf]

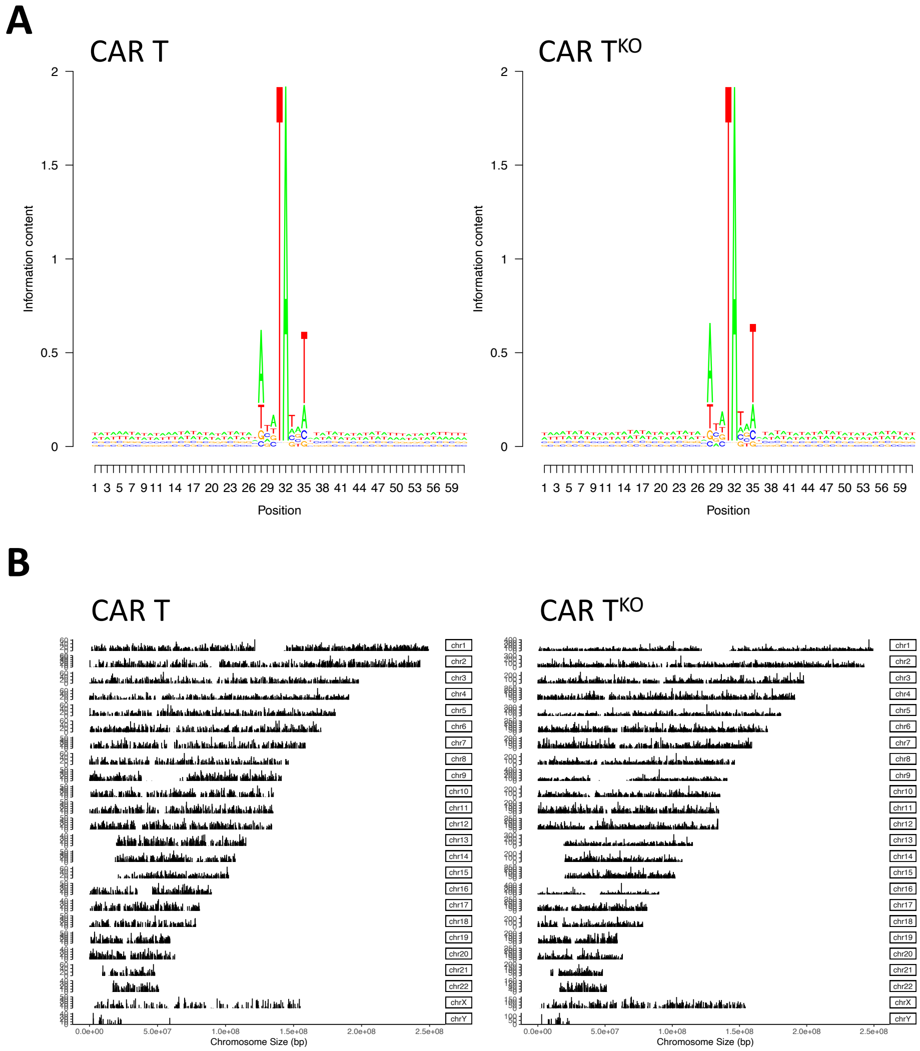

Fig. S10. Safety analysis of HLA-I^{KO}/TCR^{KO} CD33-CAR-T cells. (A) Integration site analysis of CD33-CAR-T^{KO} (n=3) and CAR-T cell (n=3) productions. **(B)** Distribution of CAR insertions within the genome of CD33-CAR-T^{KO} and CAR-T cells.
